# Agri-environmental policies have reduced cropland degradation globally

**DOI:** 10.1038/s43016-026-01359-4

**Published:** 2026-05-18

**Authors:** Guyo Dureti, Hadi Hadi, David Wuepper

**Affiliations:** Land Economics Group, Institute for Resource and Food Economics, University of Bonn, Bonn, Germany

**Keywords:** Economics, Environmental impact, Environmental economics

## Abstract

Cropland degradation is a major threat to agricultural production and the environment. To address this, countries around the world implement a range of public policies. We used global satellite remote sensing data (~83 million measurements of cropland condition from 2001 to 2019) and 2 complementary quasi-experimental impact evaluation methods (difference in discontinuities and difference in differences) to assess the impact of these policies on the state of the world’s cropland. We found that, on average, these policies have improved cropland globally by at least 2% and possibly up to 5%. Country-level heterogeneities reveal effects ranging from 0 to over 20%. This heterogeneity is explained by differences in countries’ institutional and governance characteristics and policy budgets. Moreover, by estimating variation in policy intensity across policy types, we found that agri-environmental payments and soil and land-use regulations were most effective, yielding estimated improvements of 0.8% and 0.9% per additional policy.

## Abstract (web summary)

Evidence on the effectiveness of national policies against cropland degradation is limited. Using global satellite data from 2001 to 2019, this study addresses this gap and reveals which country characteristics are associated with distinct policy success levels.

## Main

Humanity relies on land for over 95% of its food production, and about a quarter of wildlife finds its habitat on land^1,2^. However, most of the world’s arable land is affected by land degradation^3,4^, and the global rate of agricultural soil loss far exceeds the natural rate of soil replenishment^5,6^. According to a recent report from the United Nations (UN) Food and Agriculture Organization (FAO), one third of the world’s soils have suffered degradation^7^. In monetary terms, the annual global economic cost of soil erosion by water alone is estimated at US$8 billion, with a concomitant loss of 33.7 million tons of food production^8^.

To make agriculture more sustainable, countries around the world have designed and implemented several public agri-environmental policies over the past few decades^9,10^. Yet, it remains unclear whether and under which conditions these policies have been effective, both globally and across countries—not because of a lack of scientific and policy interest^11–13^ but because existing research has been limited to case studies. For example, in some African countries, such as Uganda and Ethiopia, studies have shown that governmental and non-governmental agricultural policies have reduced cropland degradation^14–17^. In Europe, Wuepper^18^ found that European Union policies have reduced soil erosion over the past decade, but with considerable spatial heterogeneity. In China, Tang et al.^19^ showed that farmland conservation policies improved cropland conditions. In the USA, Ayres et al.^20^ found that improved water rights increased the value of agricultural land in California, while Deines et al.^21^ and Chen et al.^22^ showed that long-term conservation and non-till adoption improved soil conditions and farmland values. In Brazil, Polidoro et al.^23^ found that agri-environmental policies reduced soil erosion.

Besides geographic evidence gaps in the literature, there is also a possible publication bias, as studies tend to examine successful policies rather than unsuccessful ones. The full global picture—in terms of both the average effect of public policies on croplands and the distribution of more and less successful policies—is currently lacking. The ability to bridge this gap has been hampered so far by inadequate data availability (measuring land degradation and policies at the global level over time), methodological limitations (identifying policy effects at such a large scale) and computational power^24^.

**Fig. 1:**
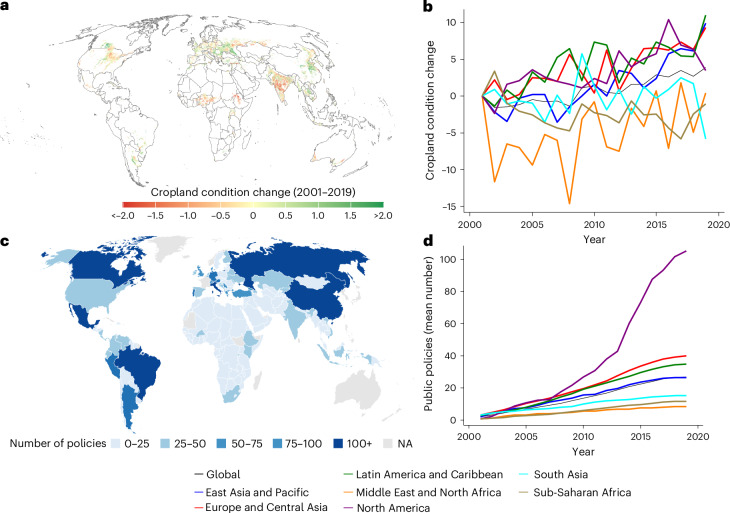
Spatial and temporal variation in cropland condition and public policies (2001–2019). **a**, The standardized global distribution of the change in cropland condition between 2001–2003 and 2017–2019 based on 10 × 10 km^2^ pixels. The values are expressed as *z*-scores relative to the global distribution, with lower values (red) indicating poorer condition and higher values (green) indicating better condition. **b**, Temporal variation in mean cropland condition by FAO region. **c**, The total number of public policies adopted by each country, with some countries implementing only a few policies, while others adopted over 100. NA refers to countries with no identified relevant public policies. **d**, The temporal evolution of the policy numbers in **c** by FAO region. The map in **a** is based on approximately 253,274 cropland pixels (~5 million pixel–period observations). See Extended Data Fig. 1 for both standardized and percentage change in cropland condition. Basemap data from Natural Earth (https://www.naturalearthdata.com). Source data

Here we empirically quantify the effect of countries’ public policies on their croplands. We use a recently published global database of public policies^10^ with a satellite-based measure of cropland degradation and improvement to examine whether these policies translated into changes in the condition of croplands in subsequent years (Fig. 1). For this, we use two complementary quasi-experimental impact evaluation methods with about 83 million cropland condition measurements worldwide over the period 2001–2019.

Our measure of cropland condition is defined as a long-term trend in the biological productivity of cropland after agricultural inputs and climatic influences are removed. To measure this at the global scale, we leveraged recent advances in satellite data accessibility and cloud-based computing platforms, which enhanced the feasibility of cropland examination at fine resolution and over long time periods^16,25,26^ at the global level.

We specifically followed a three-step process to construct a measure of cropland condition. First, we identified global cropland areas using the information provided by Sulla-Menashe et al.^27^. From these areas, we quantified the annual crop productivity at 1 × 1 km^2^ grid resolution, using readings of the annual maximum of the enhanced vegetation index (EVI)^28^. By itself, this productivity measure captures the sum of various factors, including agricultural inputs and agro-climatic conditions. To remove the influence of these non-cropland factors, we residualized the productivity measure by controlling for climatic variables and agricultural inputs (see Methods for the details). The obtained measure of cropland condition is gap-free at the global scale, at a fine resolution of 1 × 1 km^2^, from 2001 to 2019 (see Fig. 1a,b and ‘Quantifying cropland condition’ in Methods for the details).

It is well known that different contextual factors matter in enabling public policies to achieve their objectives, including regulatory environments, resource endowments, state capacities, political interests and institutional quality, among others^29–31^. To measure countries’ policies, we began with a simple cumulative count of these policies over the past two decades (2000–2019). A concern with this approach is that it only works if the number of policies is a reliable measure of overall policy strength. If it were common for some countries to implement many small and ineffective policies and for others to implement one large and effective policy, then the count of policies would not be a reliable policy measure. However, there is no common real-world example of such a pattern. Across the world, the political set-up is such that the countries that implement large and effective policies generally are also the countries that implement a larger number of policies, and vice versa^32,33^. For this reason, the number of policies also captures the overall policy effort and ambition (Supplementary Fig. 1).

As an alternative policy measure, we extended our approach in two complementary ways. First, we constructed several augmented policy indices by weighting the number of implemented policies with relevant contextual variables, taken from the World Bank’s Worldwide Governance Indicators (WGIs) and the International Monetary Fund’s Environmental Protection Expenditures (EPE)^34,35^. Second, we used a policy measure that assigns each country to the treatment group if it has at least one policy and to the control group otherwise. This measure allows us to explicitly examine trends in cropland conditions during the pre-policy period. Across all these alternative measures, we found consistent patterns of policy effects (see Methods for the details).

Our empirical analysis employed two complementary state-of-the-art econometric techniques: difference in discontinuities and difference in differences. First, we focused on the trend of cropland condition near international borders to quantify how the discontinuities in cropland condition changed from before to after the implementation of policies on either side of a border. Because cropland conditions tend to be similar in close geographical proximity, the abrupt division of the land into different countries helps disentangle the influence of countries from natural and climatic conditions, while the change over time helps separate the influence of specific policy implementations from broader country characteristics such as income, population and politics^36,37^. A drawback of this approach is the focus on just border areas, which may not be fully representative of the rest of the country as a whole. We therefore complemented this approach with an empirical strategy that exploits data from the entire countries^38^. We computed the average cropland condition for each country and year and then linked this to countries’ policies, assessing relative changes among neighbouring countries over time. We implemented this twice—once statically and once dynamically. The latter takes into account the staggered adoption of the policies^39,40^. Finally, we analysed impact heterogeneity in two dimensions. First, we performed country-level heterogeneity analyses to assess the geographic variation in policy effectiveness and identify where in the world these policies have been most successful. Second, we examined policy-type heterogeneity by categorizing policies into four groups (that is, soil and land-use regulations, input regulations, habitat and biodiversity regulations, and agri-environmental payments). Here we applied heterogeneity-robust difference-in-differences with a continuous treatment design to estimate the marginal effect of each type of policy.

## Results

We begin by presenting the main findings of the two quasi-experimental impact evaluation approaches we employed. The first is a border-region-based analysis. The second is based on entire countries. At the end, we present results from two heterogeneity analyses for variation in effects across countries and across different policy types.

### The impact of public policies on cropland condition

**Fig. 2:**
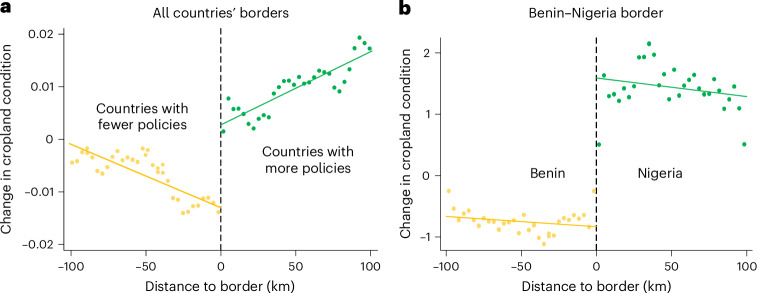
Border discontinuities in cropland condition. **a**, The spatial variation in cropland condition within 100 km of international borders using ~83 million grid-cell–year observations (measured as the annual residualized maximum EVI at 1 × 1 km^2^ resolution). The distance to the border represents the normalized distance of the grid pixel in kilometres running from the border points on both sides. The negative distance on the left represents countries with fewer public policies, while the positive distance on the right represents countries with more policies. Yellow and green dots indicate local average values of the cropland condition measure, and yellow and green lines indicate the fitted spatial trends. The dashed vertical line is the average global border. **b**, Similar distribution plots as in **a**, but only along the Benin–Nigeria border, where Nigeria has more public policies than Benin. Both graphs highlight the considerable difference in cropland condition across countries with more and fewer cropland-related policies. Moreover, countries with few public policies appear to be on a negative trend of cropland condition (below-zero values on the *y* axis). See Extended Data Fig. 2 for additional examples. Source data

When we focus only on countries’ borders, there are sharp discontinuities in inter-annual changes in the condition of the cropland right at the borders, even though the environmental growing conditions are commonly similar in such geographically close areas (Fig. 2). This global average cropland condition around international borders was constructed by aggregating all individual country border pairs around the world. As a specific example of one of these borders for illustration purposes, we observed a sharp discontinuity in cropland condition between Nigeria, which has more public policies, and Benin, which has fewer policies (Fig. 2).

**Fig. 3:**
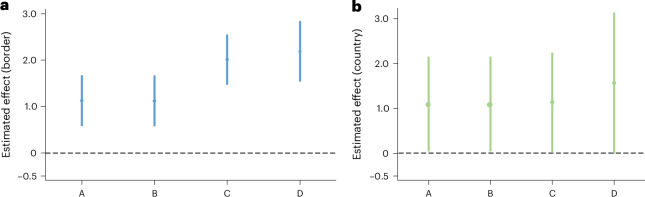
Main policy effect estimates. **a**, Border-level regression results with full controls (that is, distances, coordinates, gross domestic product (GDP), share of agriculture in GDP, HDI, corruption, rule of law, political stability, accountability, environmental expenditures, border and year fixed effects). **b**, Country-level regression results with full controls (that is, GDP, share of agriculture in GDP, HDI, corruption, rule of law, political stability, accountability, environmental expenditures, country and year fixed effects). In both panels, uppercase letters (A–D) indicate different policy measures: policy variable without weight (A), environmental expenditure weighted policy (B), government effectiveness weighted (C) and the index of all weighted policy variables (including environmental expenditure, government effectiveness, regulatory effectiveness, corruption control, and accountability and transparency measures) (D). For **a**, the sample comprises ~83 million grid-cell–year observations at 1 × 1 km^2^ resolution from country border areas. For **b**, it comprises country–year averages from entire countries (beyond border areas) for 160 countries over 2001–2019 (*n* = 3,040). The error bars represent 95% confidence intervals. Source data

Our first empirical strategy to identify the impact of countries’ public policies on their cropland condition exploits exactly these border discontinuities that are visible in Fig. 2. The spatial difference in discontinuities design consists of two main steps^37^. First, an optimal bandwidth around the borders is estimated, which balances a trade-off between bias and variance^41^. According to our analysis, the optimal bandwidth is a 26-km border radius. This implies that within this 26 km, both sides of the average border around the world are sufficiently similar in terms of growing conditions (climatic conditions), which allows an unbiased identification of the effect of countries’ policies. In the second step, on the basis of the optimal distance, the policy impact is estimated to identify how the border discontinuities change from before to after the implementation of public policies (Fig. 3a).

Figure 3 shows the findings from two alternative measures of public policy variables. The first measure is based on a simple cumulative count of policies implemented by the countries over the period 2000–2019 (Fig. 3a (A)). The second measure is based on weighting the number of implemented policies by the country characteristics that are likely to influence policy design and implementation^10^. Overall, the estimated effect of the public policies averages around 2%, when the policy variable is weighted by different indicators and all relevant controls are added (Fig. 3a (B–D)). This result implies that public policies have improved cropland condition on average by approximately 2% in countries with at least one more policy compared with countries with fewer policies. This estimated effect represents the average effect of having at least one more policy relative to neighbouring countries.

To examine the extent to which the above border-discontinuity estimates are representative of the impacts in the countries at large, we performed complementary analyses using a difference-in-differences framework that is not restricted to border areas. Here we incorporated all cropland pixels within each country by aggregating all cropland pixel data points to the average country level and then estimated a generalized difference-in-differences as an alternative identification. Our empirical specification closely mirrored that of the border-discontinuity analysis, except that we now considered the whole country instead of being limited to within 100 km of the country’s border areas. Crucially, we did not compare all countries globally; instead, we preserved the spirit of the border design by comparing countries to their immediate neighbours. This ensured that treated and control units remained regionally comparable, while allowing us to assess whether the locally identified border effects generalize beyond border areas. In this sense, the country-level analysis addresses a key limitation of border designs—limited external validity—while the border analysis mitigates concerns about unobserved heterogeneity that often arise in cross-country comparisons. Using the difference-in-differences approach, we found that public policies improved global cropland condition on average by about 1.5% (Fig. 3b). These results are consistent with our border area estimates, albeit a bit lower. As in the difference in discontinuity designs (Fig. 3a), we controlled for various country characteristics as well as country and year fixed effects, and we clustered standard errors at the country level.

**Fig. 4:**
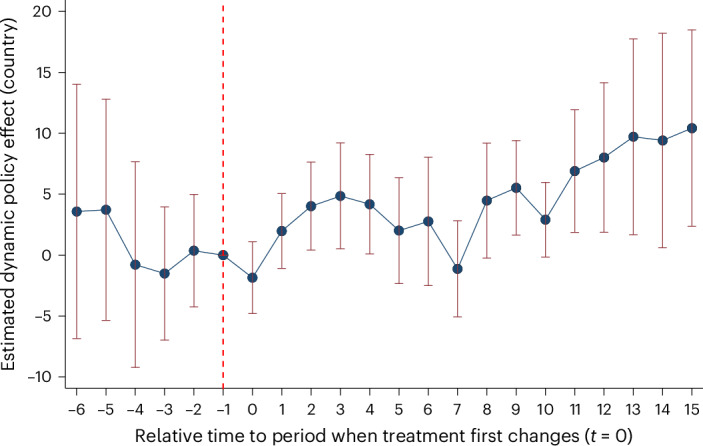
Dynamic policy effect estimates. Regression results using the staggered event study estimator. Estimates are relative to changes from the first period. Policy effects prior to implementation are insignificant, supporting the parallel trends assumption. Post-implementation, effects are significant except for some dips in the seventh year. Overall, public policies improved cropland condition by an average of 5% over the past 2 decades. The sample size (*n* = 3,040) corresponds to country–year observations, which are biological replicates. Each observation is a country–year average of cropland condition; multiple grid-cell measurements within a country–year were averaged (technical replicates). Treated units are country–years during which relevant policies were active; control units are country–years without active policies. The red dashed line refers to the reference period. The error bars represent 95% robust confidence intervals around the mean estimates. Source data

As an alternative approach to the country-level analysis, we estimated a dynamic event-study-based difference in differences^39,42^. For this, we modified our treatment variable from a cumulative policy measure to one that assigns each country to the treatment group if it has at least one public policy and treats it as a control otherwise. This finding confirms that the effects of public policies on cropland condition are all consistently pointing in the same direction. While the policy effect varies from year to year (as indicated by the event study coefficients), the average effect across all post-treatment event times is approximately 4.4% (see Fig. 4 and Supplementary Table 1 for further details). Moreover, we can also clearly observe from Fig. 4 the absence of the policy effects in the pre-policy period.

### Estimates of the heterogeneity effects of public policies

The policy effects discussed so far provide an overall picture of all public policies for all countries, where our estimates are based on the binary measures of policy. However, our results mask two important heterogeneities: (1) variation in policy intensity across policy types and (2) spatial variation in policy effectiveness across countries. Here we extend our analysis in these two dimensions.

**Fig. 5:**
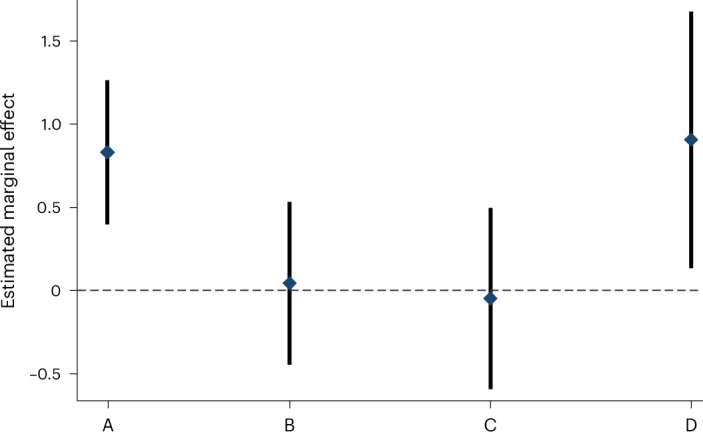
Effect estimates by policy type. Regression results for four policy types: soil and land-use regulations (A), input regulations (B), habitats and biodiversity regulations (C) and agri-environmental payments (D). Estimates were obtained using the heterogeneity-robust difference-in-differences design with a continuous treatment estimator and are plotted with 95% robust confidence intervals (see the corresponding event study results in Extended Data Fig. 3). The sample size (*n* = 3,040) corresponds to country–year observations, which are biological replicates. Each observation is a country–year average of cropland condition; multiple grid-cell measurements within a country–year were averaged (technical replicates). Treated units are country–years during which the relevant policy type was active; control units are country–years without active policies. The error bars represent 95% robust confidence intervals around the mean estimates. Source data

First, we estimated heterogeneous treatment effects across policy types using a continuous treatment design, which addresses concerns regarding the interpretability of the binary treatment and the loss of information on policy intensity. Specifically, we implemented a heterogeneity-robust difference-in-differences framework in which treatment intensity is measured as the number of policies within each policy category, allowing us to estimate the marginal effect of an additional policy. For this, we classified individual policies into four main groups: (1) soil and land-use regulations, (2) input regulations, (3) habitat and biodiversity regulations and (4) agri-environmental payments. Our findings reveal substantial heterogeneity in marginal effects across policy types (Fig. 5). As shown in Fig. 5, agri-environmental payments emerged as the most effective policy types, improving cropland condition by approximately 0.91% per additional policy. These payment schemes typically reward farmers for adopting more sustainable farming practices or for achieving measurable outcomes, such as on-farm biodiversity, and sometimes for both adoption and outcomes combined^43,44^. These policies offer several advantages, including that they compensate farmers financially for the costs of farming more sustainably, which can also contribute to making the entire policy mix more acceptable for the farmers. So far, however, the empirical evidence was rather mixed, and different studies have found that for some practices and in some countries, past payments have not always caused a change in practices (the payments had limited ‘additionality’).

We also found that soil and land-use regulations led to similarly strong marginal effects, with an estimated improvement of about 0.83% per additional policy. This group includes policies that directly aim to improve the sustainability of farm management practices, such as reducing soil degradation (for example, erosion and compaction), and tenure security and property rights to encourage long-term investment in agricultural land.

In contrast, we found that input regulations, policies aimed at reducing fertilizer and pesticide use, did not have a statistically significant impact on cropland condition. It should be noted that agricultural management is controlled for in all our specifications, which might limit our ability to detect direct effects via changes in input use. Nevertheless, we could pick up indirect effects if farmers adopted agro-ecological practices in response to input regulations, which could, in turn, improve cropland conditions. While the estimated coefficient suggests potential indirect effects, the large standard errors may point to substantial heterogeneity, making it difficult to precisely estimate these effects. The related literature offers limited evidence about the impact of input regulations on cropland, except Tang et al.^19^, who investigated the effect of an action plan aimed at protecting black soil farmland in North China.

We also found a near-zero, statistically insignificant effect of habitat and biodiversity regulations. These policies are especially common in Europe but usually do not directly target croplands, although such policies sometimes encourage the creation of landscape structures (such as hedgerows, wildflower strips or living fences) to support biodiversity.

In addition to the heterogeneity in policy intensity, we examined the spatial heterogeneity in policy effectiveness at the country level to further identify where in the world these policies are more or less effective in improving cropland condition. For this analysis, estimated border discontinuities were aggregated at the country level to obtain the average estimate for each country. While this dimension of heterogeneity does not reflect variation in treatment intensity, it provides insight into cross-country differences in effectiveness that may reflect their institutional quality, enforcement capability, agro-ecological conditions or implementation quality.

**Fig. 6:**
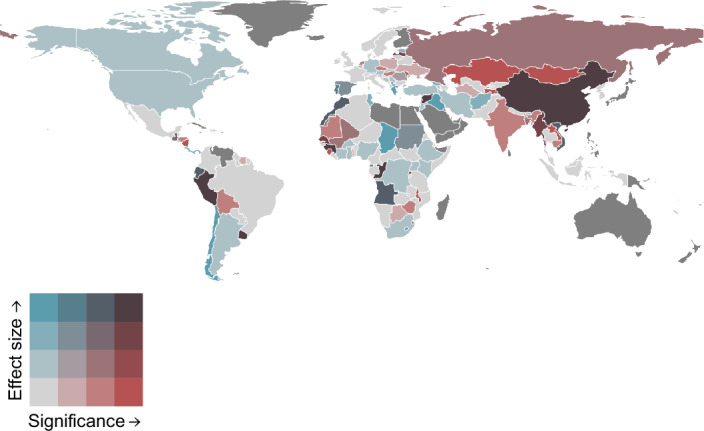
Country-level policy effect estimates. Regression results from the estimated effect of public policies per country. The effects are estimated relative to all neighbouring countries, where a country is considered treated if it has more cumulative policies than the average cumulative policies of its neighbours. The sample is taken from ~83 million grid-cell–year observations at 1 × 1 km^2^ resolution from country border areas. The estimated effects for each country are plotted on a bivariate map on the basis of the effect size and statistical significance. The effect size varies as ≤0, 0–4, 4–8 and >8 (9–22), while the significance level varies as >10%, 10–5%, 5–1% and <1% (all in increasing order). For example, the black red at the top right of the square indicates a statistically significant (at <1%) higher policy effect (>8%), while the white red at the bottom left indicates a lower policy effect (≤0) that is not significant (>10%). Basemap data from Natural Earth (https://www.naturalearthdata.com). Source data

Our results show that the effectiveness of public policies is heterogeneous across countries, ranging from negligible to beyond 20% (Fig. 6). For example, we observed strong policy effects of more than 10% in some countries in Asia (such as China), Latin America (such as Uruguay) and Africa (such as Angola). We also obtained relatively moderate policy effects of around 6% in some countries, including Rwanda, Peru, Portugal and Vietnam. However, we found statistically insignificant policy effects for some countries in Asia (such as Mongolia and India), Latin America (such as Bolivia and Nicaragua), Africa (such as Botswana and Zimbabwe) and Europe (such as Ukraine). This finding highlights considerable heterogeneity in the relative effectiveness of policies across countries.

**Fig. 7:**
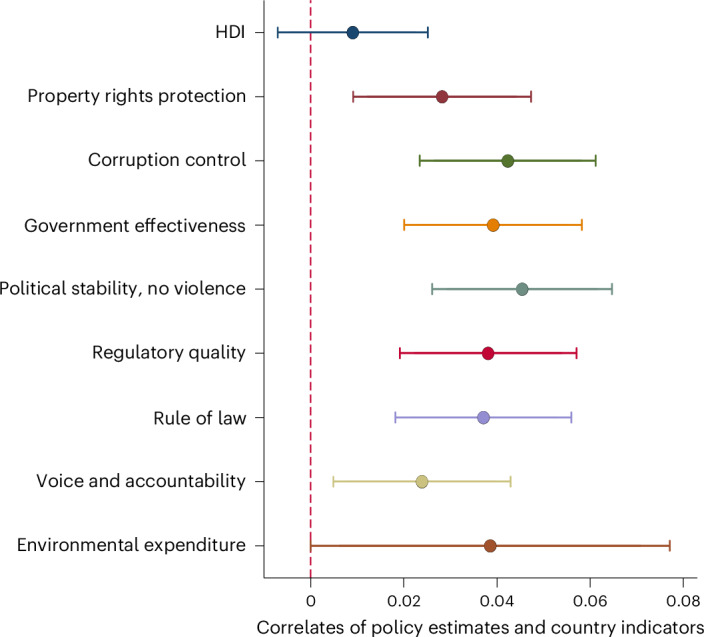
Underlying conditions for the effectiveness of public policies. Coefficient plots of the probability of policy effectiveness based on various standardized country-level indicators. The filled circles indicate point estimates along the *x* axis, and the horizontal error bars show 95% confidence intervals around the mean estimates. Each indicator is related to the probability of policy effectiveness, defined as the individual country-level policy effect obtained from the difference-in-discontinuities regression. These indicators are tested as mediated factors for policy success in improving cropland condition. The sample size (*n* = 3,040) corresponds to country–year observations, which are biological replicates. Each observation is a country–year average of cropland condition; multiple grid-cell measurements within a country–year were averaged (technical replicates). Source data

Beyond cross-country and policy-type heterogeneity, we also assessed under which conditions these policies are more or less successful in general. This is to provide insights into the relevant policy contexts that matter for the success of public policies. In other words, we attempted to identify what common pattern we observe for countries with more successful policies. We used nine indicators, including the human development index (HDI), property right protection, control of corruption, government effectiveness, political stability, regulatory quality, rule of law, voice and accountability, and environmental expenditure. Our findings show that all these indicators are positively and significantly (except for HDI) correlated with the probability of policy effectiveness. These results suggest that a one-standard-deviation increase in each of these indicators is associated with a 3–5% increase in the probability of policy effectiveness (Fig. 7).

## Discussion

As we reach halfway into the UN Decade on Ecosystem Restoration (2021–2030), understanding the conditions under which policies are most effective remains a top priority to ensure that countries implement the most efficient and impactful measures possible. We found that, overall, countries’ agri-environmental policies lead to a considerable improvement in global cropland condition. At the same time, we observed considerable heterogeneity in policy effectiveness across policy types and across countries.

**Fig. 8:**
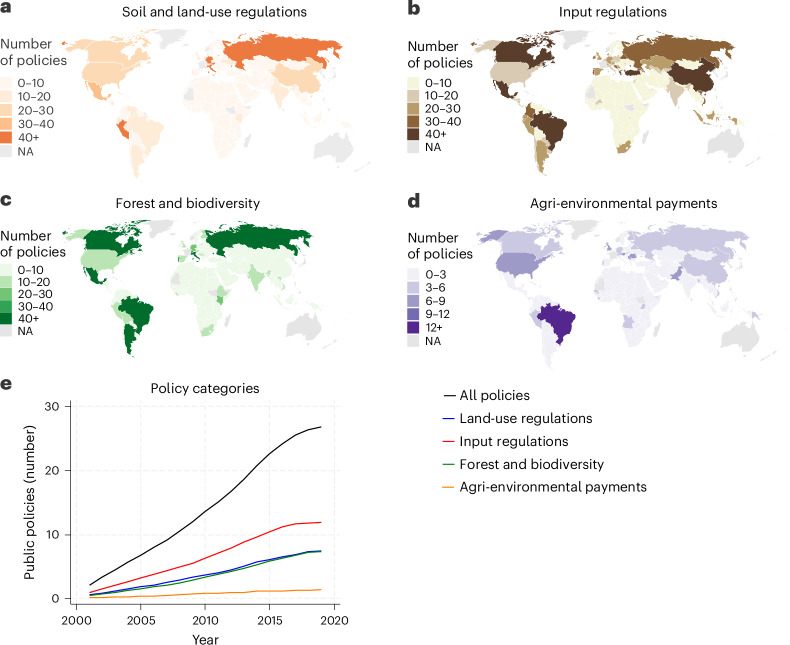
Distribution of four categories of public policies. **a**–**d**, The distribution of four categories of public agri-environmental policies at the country level: soil and land-use regulations (**a**), input regulations (**b**), forest and biodiversity policies (**c**) and agri-environmental payment policies (**d**). In **a**–**d**, each map reports the cumulative number of policies of each type adopted by countries over the period 2001–2019. In the maps, darker colour intensities indicate countries with higher policy counts, while lighter intensities indicate fewer policies. **e**, The year-to-year variation in the number of policies across the four categories. Basemap data from Natural Earth (https://www.naturalearthdata.com). Source data

Among the four policy categories (Fig. 8), agri-environmental payments and soil and land-use regulations showed the strongest positive effects on cropland condition, reflecting the role of instruments that directly influence land management practices and provide clear incentives for sustainable production. In contrast, input regulations and habitat and biodiversity policies showed no significant impact, highlighting their more limited or indirect links to cropland outcomes. Overall, these findings highlight that not all agri-environmental policies are equally effective in improving cropland condition, and targeted policy design, especially policies addressing land use and soil management directly, may offer the highest returns.

We also observe considerable cross-country heterogeneity, with estimated effects ranging from 0 to over 20%. This variation is systematically explained by countries’ institutional characteristics, such as political stability, corruption control, government effectiveness and regulatory quality, as well as differences in policy budgets. Countries with stronger institutions tend to implement more impactful agri-environmental policies, suggesting that institutional capacity shapes both policy design and implementation. We also drew some descriptive spatial and temporal patterns from our new measure of global cropland condition. Across regions, we observed widespread degradation in sub-Saharan Africa, parts of South Asia and the Middle East, while considerable improvements have occurred in Eastern Europe, Central Asia and parts of Southeast Asia. Moreover, countries with better cropland conditions exhibit stronger overall agricultural performance measured in terms of yield or total factor productivity (TFP), supporting the relevance of cropland conditions as a proxy for sustainable land-based productivity.

These insights hold important implications for multinational international initiatives such as the UN FAO’s Global Soil Partnership, the UN Decade on Ecosystem Restoration, the Sustainable Development Goals and similar global initiatives that aim to further harmonize efforts across nations towards the sustainable management of land resources worldwide. In particular, our analysis enables the benchmarking of policy performance at the country level in such a way that it is possible to assess the potential effectiveness of each country’s policies relative to its neighbours. This gives an essential implication of what good public agri-environmental policies can be learned from the best-performing countries to improve policymaking in lower-performing countries. For example, China, one of the leading examples, has implemented over 100 cropland-related policies over the past two decades, which have also been shown to be effective in improving cropland condition^19,45^. However, many neighbouring countries lag behind in policy performance. Our findings suggest that countries can benefit from learning from such examples to improve their own policies and land conservation efforts.

Before concluding, we acknowledge a few limitations of this study that also point to opportunities for future research. First, while the study leverages the most recent globally consistent satellite datasets currently available, some constraints remain. Irrigation, for example, was measured only twice (in 2005 and 2010), yet irrigation levels probably change over time and may be correlated with policy activity and economic development. However, this is likely to have limited implications for our analysis, as irrigation is typically a fixed form of infrastructure and thus less variable than other inputs such as fertilizer. Moreover, its influence can be at least partly captured through country and year fixed effects or proxied by broader indicators of economic development. Second, we recognize that without exhaustive policy data across all domains and countries, it is not feasible to cleanly isolate the effects of policies from those of other co-occurring policies or previous policies that may differently affect post-treatment outcomes. While our analyses employed a broadly representative policy database, different empirical designs and robustness checks, future studies can build on our findings to address potential remaining challenges.

Third, we acknowledge that the relationship between vegetation indices such as EVI and above-ground biomass is not strictly linear and may vary with phenological timing^46,47^. While this nonlinearity is less problematic in croplands than in dense vegetation, we did not interpret a given percentage change in EVI as an equivalent percentage change in biomass. Instead, we used relative changes in residualized EVI as a proxy measure for relative changes in cropland condition^48^. Fourth, due to data constraints, we did not assess the cost-effectiveness of these policies. Lastly, while we used the most recent globally consistent policy and satellite datasets available at high spatial resolution, future research could also build on this work by assessing how evolving smart farming technologies, climate pressures and institutional reforms continue to shape the effectiveness of agri-environmental policies.

In conclusion, we present a global picture of the current performance of agri-environmental policies around the world. By documenting both country- and policy-type heterogeneities and by identifying institutional and design features that may matter for success, our analyses offer a road map for more targeted, effective and context-sensitive policies to sustain and restore global cropland. In doing so, we provide new comprehensive evidence to support national and international efforts by linking global empirical findings to actionable, policy-relevant insights. These findings contribute not only to scientific understanding but also to real-world decision-making for more effective and efficient policy design and implementation, ensuring the long-term sustainability of the world’s cropland.

## Methods

### Quantifying cropland condition

In this study, we defined cropland condition as a trend in biological productivity, measured using the EVI net of agro-climatic factors and agricultural inputs. A growing number of studies use remotely sensed data such as the EVI or normalized difference vegetation index to measure land degradation^16,43,49,50^. A negative trend in these indicators may suggest land degradation, while a positive trend may suggest land improvement, depending on how well other driving factors are taken into account. Vegetation indices are influenced by various factors, including natural climatic dynamics (for example, rainfall, temperature, solar radiation and topography) and anthropogenic factors (for example, the application of fertilizer, agro-chemicals, irrigation and tillage). This is particularly important for cropland since crop farmers may apply more fertilizer or invest in irrigation to compensate for worsening cropland conditions. Increasing investments without crop yield improvements can thus indicate land degradation, as can declining crop yields without increasing investments. In this circumstance, to interpret EVI trends in terms of land degradation or improvement, it is vital to remove the confounding influence of the climatic and human activities^16,43,51^. This is now feasible even on a global scale with the increasing availability of consistent environmental and agricultural variables. We leveraged this to construct our measure of cropland condition outcome in the following ways. First, we identified cropland pixels at the global scale using annual land cover maps^27^. Second, we quantified the maximum crop productivity of each year at a resolution of 1 × 1 km^2^ grid cells, using readings of the maximum of the EVI^28^. Third, we statistically removed the above-discussed masking factors using the following equation:

1 $${S}_{{it}}={{\bf{\uptheta}}} {C}_{{it}}+\gamma {M}_{{it}}+{\mu }_{{it}}$$

where *S*_*it*_ represents the logarithm of the annual maximum EVI for pixel *i* in year *t* at a resolution of 1 × 1 km^2^ grid cells; *C*_*it*_ stands for climate variables, including precipitation^52^, temperature^53^, solar radiation^54^ and topography^55^, and their respective interaction terms; **θ** is a vector of coefficients on climate variables; *M*_*it*_ is for human management practices, including fertilizer^56^, irrigation^57^ and the share of crop pixels cultivated^27^, and their respective interaction terms; and *μ*_*it*_ is the error term.

To obtain a measure of cropland condition from equation (1), we first regressed pixel-level measurements of EVI on the above indicators of climate and human management variables. We then predicted the residual, which left us with the EVI trends that reflect the cropland condition, our measurement of interest. While this measure may not directly capture every dimension of ecological health, such as soil biodiversity or chemical composition, it moves beyond management inputs and climate-driven yield proxies to offer a more intrinsic indicator of land condition performance. We followed a similar procedure for both the border-region level and the country-level average values of cropland condition. Regarding management practices, it can be argued that public policy may influence the management practices themselves, through which it then affects the land condition outcome of interest. To understand the extent of this, we performed additional analysis without controlling for management practices. We found relatively higher policy effects, which implies that it is important to control for these management variables (Supplementary Fig. 3).

Figure 1a presents high-resolution maps at 10 × 10 km^2^ pixel levels to capture heterogeneity within countries and across time on the basis of the spatial and temporal richness of the pixel-level data. To construct this map, we took average values over three-year windows (2001–2003 and 2017–2019) to minimize potential variability and possible year-specific spikes that may not be representative, or missingness of data due to the cloud cover for a specific pixel in a given year or possible measurement errors. The figure reveals substantial spatial heterogeneity, with the largest positive changes most pronounced in North America (especially Canada), Eastern Europe, Central Asia and parts of South and East Asia, probably reflecting effective management practices, sustainable intensification or agri-environmental policies. Negative changes are more pronounced in sub-Saharan Africa and scattered regions of South America and the Middle East, probably reflecting ongoing land degradation, climatic stress or low institutional capacity. Some countries, such as India and the USA (Midwest), show mixed changes with some parts improving and others declining. These spatial dynamics highlight the importance of using high-resolution maps instead of relying solely on country-level averages, which can mask localized degradation or improvement.

The patterns we observed closely align with previous studies for several regions and countries (for example, Europe, North America, southern Africa and Sahel regions, Latin America, and Southeast Asia, including China) but deviate for others (for example, India)^48,58–62^. These differences may arise because most prior studies assess only ‘greening’ trends using direct measures of normalized difference vegetation index or EVI, without explicitly accounting for climatic and management effects. In such cases, observed greening or browning may be driven by agricultural intensification or climate change^62,63^. Our measure accounts for these influences, capturing intrinsic land productivity. Overall, these global patterns highlight both progress and persistent challenges in cropland sustainability and provide a descriptive foundation for our policy impact analysis.

In Fig. 1b, we illustrate spatiotemporal variation in cropland condition relative to an early-period baseline across global regions. The global trend shows gradual improvement with modest overall recovery in cropland condition worldwide. However, at the regional level, trajectories reveal several heterogeneities. For example, North America, East Asia and Pacific, Europe and Latin America exhibit steady improvement in cropland condition over time, with some fluctuations but a broadly positive trajectory. In contrast, sub-Saharan Africa, the Middle East and South Asia mostly show persistently deteriorating conditions with considerable fluctuations, highlighting the dynamic nature of cropland condition in areas facing environmental and socio-economic pressures. Overall, this regional breakdown highlights areas with persistent vulnerabilities and consistent improvement beyond static global averages.

Finally, we explored the relationship between cropland condition measures and two key agricultural performance indicators: TFP and yield. We found strong positive associations between cropland condition and both yield and TFP across countries (Supplementary Figs. 4 and 5). In particular, countries with better cropland condition tended to exhibit higher average yields and TFP growth, suggesting that our measure captures meaningful variation in agricultural performance. However, this relationship is not uniform for all countries, especially for yield. For example, in countries such as Ethiopia, we observed a substantial increase in yields over time, while cropland condition has remained flat or declined (Supplementary Figs. 4 and 5). These divergences may suggest that yield improvements in some contexts may be driven by input intensification or short-term factors^64^, rather than sustainable improvements in land quality. Overall, these patterns support the validity of cropland condition as a proxy for land-based productivity improvements, net of climatic variability and external input intensification.

### Quantifying public policies

With regard to public policies, we focused on the government-led agri-environmental policies implemented within the administrative boundaries of countries^10^. These policies are of different types, ranging from legislative command-and-control measures to payment-based policies and various combinations of frameworks and monitoring policies (see Supplementary Table 2 for example policies). Moreover, while some of these policies directly aim to improve cropland condition (for example, soil erosion control or fertilizer regulation for soil health), others are more indirectly relevant (for example, biodiversity or forest conservation) (Supplementary Table 2). For the policy heterogeneity analysis, we categorized them into four groups: (1) soil and land-use regulations, (2) input-related policies (for example, fertilizers and agro-chemicals), (3) forest and biodiversity policies and (4) agri-environmental payment schemes (Fig. 8). While the policy database may not be exhaustive, it aims to comprehensively cover the most relevant policies over the period considered (Supplementary Fig. 6). We considered about 4,700 public agri-environmental policies implemented all around the world during the period 2001–2019.

Measuring these policies is not trivial due to the absence of a widely accepted policy indicators. The major limiting factors are multi-dimensionality in policies (multitude of instruments, exemptions, phase-in periods and accompanying measures), identification issues (the ability to attribute observed changes to actual policies instead of other factors) and data coverage and availability (including the tedious process of collating policy documents), inter alia^65,66^. To overcome this, existing research usually relies on some outcome-based data, such as pollutant emission data^67,68^, or the sheer number of policies to construct a proxy measure^32,69^.

Here we began with a simple cumulative count of the policies that countries have implemented over the past two decades (2000–2019). While counting the number of policies may not be a perfect measure of policy, it can be a surprisingly good indicator of countries’ ‘policy effort’. For example, it is rare for a country to implement just one very large and ambitious policy; rather, countries often design and implement a bundle of many different policies, targeting different actors and regions (for example, see ref. ^33^ for Brazil’s forest policies). In addition, countries may make many changes to their policies to achieve their goals (for example, see ref. ^32^ for climate policies). In our analysis, we have shown that the number of public policies is also strongly correlated with other policy-relevant indicators, such as budget, implementation abilities and human development required for policy design and implementation, implying that the number of policies matters (Supplementary Fig. 1).

However, this approach has clear limitations, as not all policies are equal in terms of coverage, budget or implementation process. To further improve our measurement of policies, we used two alternative policy measures. First, we constructed several policy indices by using the World Bank’s WGIs and the International Monetary Fund’s EPE^34,35^. The WGIs are proxies for countries’ implementation capacity and institutional characteristics, while the EPE implies their policy budgets. We first normalized each WGI and EPE indicator to a scale of 0 to 1 and used them as weights for the policy variable. We then created a composite index by taking the average of all weighted policy variables (that is, the sum of policy number interacted with each indicator), as our main specification. Second, we used a policy measure that assigns each country to the treatment group if it has at least one public policy and treats it as a control otherwise. We also directly counted policies within each category (above) to capture variation in policy intensity across policy types.

### Econometric approaches

The identification of the causal effect of national agri-environmental policies comes with a formidable empirical challenge due to non-random policy implementation and various natural and anthropogenic confounding factors^37,69^. Here we describe the regression models used to deal with the identification issues faced in this study. To account for the potential confounding factors, we employed two state-of-the-art econometric methods: the border-region-based difference in discontinuities^36,37^ and the entire-country-average-based spatial difference in differences^38,39^. For the difference in discontinuities, we combined two cutting-edge econometric methods—regression discontinuity design and spatial difference-in-differences—leveraging on their respective fundamental advantages. First, in the context of regression discontinuity design, we relied on the assumption that two sides of countries’ borders are naturally comparable within the closely proximate border area—providing a typical ‘natural experiment’ setting^37,70,71^. We then linked this to the difference-in-differences approach, which assumes that countries’ public policies are implemented over a specific time period, allowing us to detect changes in cropland condition after policy implementation^37^. Combining the strengths of both methods, we employed a so-called difference-in-discontinuities design to estimate the causal effect of policies right around the international country borders^37^. The key benefit of this design is that many international borders are naturally divided into two similar parts, where close to the border, both border sides are probably environmentally comparable, making them plausible counterfactuals for each other. We estimated the following regression equation:

2 $${Y}_{{ibt}}={\alpha }_{s(i,b)t}+\beta {D}_{c\left(i,b\right),t}+{\delta }_{s\left(i,b\right),t}{X}_{i}+\gamma {Z}_{c\left(i,b\right),t}+{\varepsilon }_{{ibt}}$$

*Y*_*ibt*_ denotes the cropland condition outcome (obtained from equation (1)) at pixel *i* in year *t* along the border of *b* country pair. This is constructed from the 83 million 1 × 1 km^2^ grid-cell–year cropland condition measurements during 2001–2019. A higher *Y*_*ibt*_ implies improved cropland condition and vice versa. The term *α*_*s*(*i*,*b*)*t*_ indicates the border segment along the border by year fixed effects. The border segment is constructed by dividing the country borders into bins of narrower border segments on a half-degree grid (55 km). This allows direct cross-border comparisons not only at the borders that have patches of cropland on either side of the border but also at those with patches not necessarily close to each other. Each pixel is then assigned to the closest border point, which belongs to one of the border segments. *D*_*it*_ is the country treatment of interest, with countries with more cumulative policies taking the value 1 and countries with less cumulative policies taking the value 0 at the given border *b*. This variable is constructed for both weighted and non-weighted policy measures. *β* denotes the coefficient of interest, interpreted as the average treatment effect of the policy measure. *X*_*i*_ stands for the time-invariant spatial variables: distance to border (from each side), longitude, latitude and a cross-term. Finally, *Z*_*ct*_ represents country-level characteristics such as GDP, share of agriculture in GDP, population density, population growth, HDI, corruption, rule of law, political stability, accountability and environmental expenditures, and *ε*_*it*_ is an error term. These time-varying variables control for pixel- or country-level confounding factors. **δ** and **γ** are vectors of coefficients on *X*_*i*_ and *Z*_*c*_. A full description and sources of all variables can be found in Supplementary Table 3. As our analysis relied on difference in discontinuities, we were able to account for many time-invariant confounders^37^. By further controlling for time-varying country characteristics, we accounted for country-specific common shocks—for example, sudden changes in a country’s GDP.

Our analyses using border discontinuities in policies and cropland conditions relied on a number of important assumptions that motivated different placebo and sensitivity checks. Among the multiple robustness checks, we first estimated ‘placebo’ discontinuities by artificially shifting all borders by 5, 10 and 15 km both inwards and outwards from their original location. Our findings indicate no or less discontinuity at these placebo borders, reflecting the fact that only actual political borders are relevant (Extended Data Fig. 2). As a further robustness check, border discontinuities were estimated separately by increasing the optimal border bandwidth by 10 km (to 36 km) or decreasing it by 10 km (to 16 km). Our findings are again consistent with changes in optimal bandwidth (Extended Data Fig. 3). In addition, we performed several parallel trend tests and showed that future policies do not predict past cropland condition using policy leads. This test implies that our results are unlikely to be driven by reverse causality—that is, changes in cropland condition that led to new policies (Extended Data Fig. 4a,b).

The cropland discontinuities at international borders allowed us to focus on changes that occurred in generally similar agricultural conditions (unlike the country-level analysis). For example, near most international borders, soil types, rainfall and temperature are quite similar on each border side. However, this does not hold true everywhere, as borders sometimes follow mountain ranges, rivers and other geographic features that constitute natural discontinuities^72^. As a robustness check, we estimated a set of specifications using only observations from borders without natural discontinuities (Extended Data Fig. 5). We also performed a covariate balance test to check whether time-varying observables vary across international borders. The test result indicates insignificant differences between the control and treatment groups (Supplementary Table 5).

While this method potentially identifies the causal effect of policies, locations near the international border may not be representative of the cropland condition of the overall country. To address this limitation, we expanded beyond border areas, extracted aggregated country-level average cropland conditions and linked them with countries’ public policies. To construct a feasible counterfactual, we first sorted countries relative to their neighbours in terms of the number of policies implemented and then designated them as treated if they had more cumulative policies than their neighbours in each particular year. This enabled us to establish a plausible counterfactual among neighbouring countries with relatively similar environmental conditions. We then applied generalized difference in differences to detect the change in cropland condition attributable to implemented policies by estimating the change in cropland condition from the pre-policy to the post-policy implementation period. Here, the following equation was estimated:

3 $${Y}_{{ct}}=\beta {D}_{{ct}}+\gamma {Z}_{{ct}}+{\alpha }_{c}+{\tau }_{t}+{\varepsilon }_{{ct}}$$

Here *Y*_*ct*_ represents country-level average value of the cropland condition outcome for country *c* in year *t* (obtained using equation (1)). The sample size was determined by aggregating the annual maximum EVI from the entire country for each year from 2001 to 2019 and averaging at the country level (*n* = 3,040). *D*_*ct*_ is the country treatment of interest, where countries take value 1 if they have more cumulative policies than the average cumulative policies of all their neighbours and otherwise take value 0 for the given year *t*. *Z*_*c*_ controls for the country characteristics listed above for equation (2). Finally, *α*_*c*_ is the group (country) fixed effects controlling for all approximately fixed confounding factors that differ between countries, whereas *τ*_*t*_ is the year fixed effects, which controls for all period-specific common shocks. We estimated this model with the standard two-way fixed effects model^38^. As discussed above, our results are also not specific to border regions, but country-level difference-in-differences estimates that do not rely on borders show the same patterns that we estimated in higher-resolution areas close to countries’ borders. To further test the robustness of the obtained effects, we performed various robustness checks and placebo tests to corroborate our findings. For example, we estimated the effect of policy leads on past cropland condition outcomes to understand whether future changes in policies affect past changes in cropland condition. We found no significant coefficients, implying that the parallel trend assumption holds (Extended Data Fig. 4b). We also further controlled for dominant crop types (Supplementary Fig. 7).

To approach country-level analysis from a different empirical perspective, we also estimated the dynamic difference-in-differences method^39,42^. Here we applied two measures of policies. First, we used a general binary measure where countries are automatically assigned to the treatment group if they have implemented at least one cropland-condition-relevant public policy and remain in the control group until they have implemented at least one policy. Second, we classified policies into four types (Fig. 8) and counted policies within each category to capture variation in policy intensity across policy types. While this alternative policy measure deviates from our two previous methods, it offers us three important advantages. First, it enables us to explicitly test for the presence of the effects of policies during the pre-policy implementation period. Second, it provides important evidence that our findings are not prone to an artefact of a specific policy measure. Third, defining the treatment group on the basis of no policy instead of fewer policies might provide a more precise treatment estimate, albeit very local. We specifically used the event-study estimator by De Chaisemartin and D’Haultfoeuille^39,73^. We set up our regression model as follows:

4 $${Y}_{{ct}}=\mathop{\sum }\limits_{\tau =-q}^{-1}{\sigma }_{\tau }{D}_{c}+\mathop{\sum }\limits_{\tau =0}^{n}{\vartheta }_{\tau }{D}_{c}+\gamma {Z}_{{ct}}+{\alpha }_{c}+{\tau }_{t}+{\varepsilon }_{{ct}}$$

Here *σ*_*τ*_ and *ϑ*_*τ*_ correspond to the coefficients of the leads and lags of the treatment, respectively. All other terms are as in equation (3), apart from the treatment assignment procedure. Here we measure treatment *D*_*c*_ in two ways. First, beginning with the year 2001, each country is assigned to the treatment group if it has implemented at least one cropland-relevant public policy and remains in the control group until it has implemented at least one. In this case, the coefficient of *D*_*c*_ shows the average overall effect of policies in year-specific event-study estimates. Second, we categorized policies into four groups as discussed in section 2.2 and counted policies within each category. Here, in the spirit of a heterogeneity-robust difference-in-differences framework, treatment intensity is measured as the number of policies within each policy category, allowing us to estimate the marginal effect of an additional policy. Both country and year fixed effects are included, and standard errors are clustered at the country level. The added advantage of this method is its robustness to heterogeneous treatment effects across groups and over time periods, while still relying on the parallel trend assumption. Moreover, besides being an alternative policy measure, it allows us to explicitly test for the presence of the policy effects in the period prior to policy implementation (Fig. 8 and Extended Data Fig. 6). It further works well with unbalanced panels, wherein the outcome may be affected by the lagged treatment.

As a further robustness check, we also increased the policy threshold to at least three or more policies. This allowed us to check the sensitivity of our results to the chosen threshold levels (Supplementary Fig. 8). Another important insight we gained here is that there is a continuous yearly effect of policies on cropland condition. This provides insights into how the effect of public policies on cropland condition accumulates over time, rather than occurring immediately after policy implementation. For example, the positive changes in cropland condition caused by policies may mature slowly and take some time before being fully observable. To understand this better, we attempted to detect changes in cropland condition in response to implemented policies using policy lags. Our results reveal that the estimated policy effect steadily increases over time, which aligns with our expectation that the estimated policy effect accumulates over time (Supplementary Fig. 9).

### Reporting summary

Further information on research design is available in the Nature Portfolio Reporting Summary linked to this article.

## Supplementary information

Supplementary Information Supplementary Figs. 1–9, Tables 1–5 and Notes, including legends for all figures and tables.

Reporting Summary

Supplementary Data 1 Source data for Supplementary Figs. 1–9.

## Source data

Source Data Fig. 1 Source data, compiled in four tabs of an Excel file.

Source Data Fig. 2 Source data for Fig. 2.

Source Data Fig. 3 Source data for Fig. 3.

Source Data Fig. 4 Source data for Fig. 4.

Source Data Fig. 5 Source data for Fig. 5.

Source Data Fig. 6 Source data for Fig. 6.

Source Data Fig. 7 Source data for Fig. 7.

Source Data Fig. 8 Source data for Fig. 8.

**Extended Data Fig. 1:**
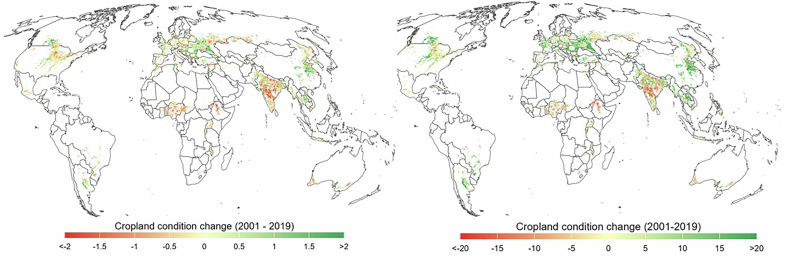
Standardized and percentage change in cropland condition. Left panel shows the standardized global distribution of changes in cropland condition between 2001–2003 and 2017–2019, based on 10 km × 10 km pixels. Values are expressed as z-scores relative to the global distribution, with lower values (red) indicating below-average changes (relative deterioration) and higher values (green) indicating above-average improvements. Right panel presents the corresponding percentage changes in cropland condition, measured as log differences scaled by 100. This measure reflects absolute changes relative to baseline conditions, with positive values (green) indicating improvements and negative values (red) indicating declines. In both panels, color intensity reflects the magnitude of change, and extreme values are truncated to enhance visual interpretability of policies across four categories. Both maps are based on approximately 253,274 cropland pixels (~5 million pixel-period observations). Basemap data from Natural Earth (https://www.naturalearthdata.com). Source data

Source Data Extended Data Fig. 1 Source data for Extended Data Fig. 1.

**Extended Data Fig. 2:**
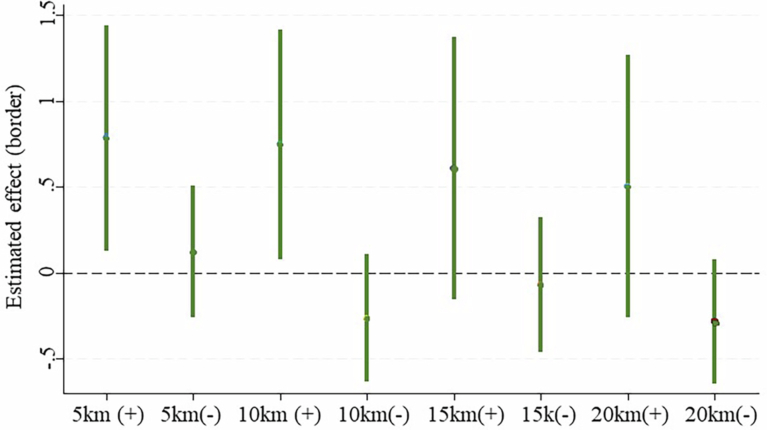
Placebo tests with artificial border (difference in discontinuities). The figure indicates the estimated effect of difference in discontinuities at different artificial borders. On the left-most side, we first increase the artificial border by 5 km in the policy side and estimate the discontinuities. Next, we estimate similar discontinuity tests with 5 km inside the less policy countryside. In the following tests, we increase or decrease the border by 10 km, 15 km, and 20 km. These different placebo tests show that the main discontinuities in cropland condition appear at the real borders, not at the artificial border. Sample size corresponds to ~83 million grid-cell–year observations. Error bars represent 95% confidence intervals. Source data

Source Data Extended Data Fig. 2 Source data for Extended Data Fig. 2.

**Extended Data Fig. 3:**
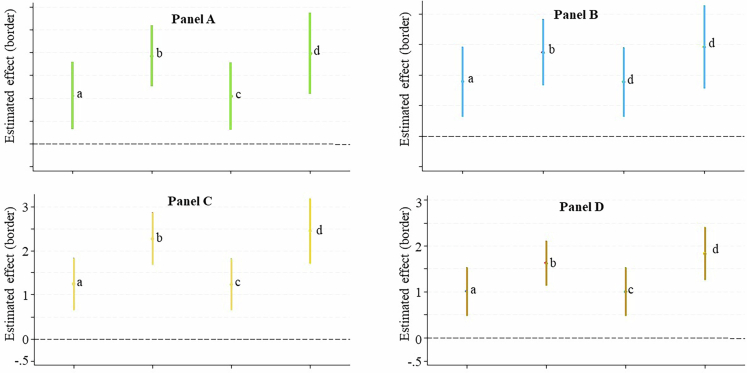
Placebo tests with bandwidth sensitivity (difference in discontinuities). Panel **a** presents the estimates of the difference in discontinuities with a border bandwidth of 16 km (by decreasing the optimal bandwidth). We also control for the distances to the border (for both sides) and coordinates (latitude, longitude, and cross-term). Panel **b** shows similar border estimates, but here by increasing the border distance by 10 km instead and making the estimation at 36 km bandwidth. Panel **c** shows a decrease in the optimal bandwidth, like in panel **a**, but we control for further country-level variables. Panel **d** shows an increase in the optimal bandwidth like in panel **b** but we control for further country-level variables. In each panel, small letters (a-i) show different policy measures: a) policy variable without weight; b) government effectiveness weighted; c) environmental expenditure weighted policy; d) average of all weighted policy variables. In general, in all these different sensitivity tests, we obtain consistent estimated effects confirming that our estimates are robust to different specifications. Sample size corresponds Source data

Source Data Extended Data Fig. 3 Source data for Extended Data Fig. 3.

**Extended Data Fig. 4:**
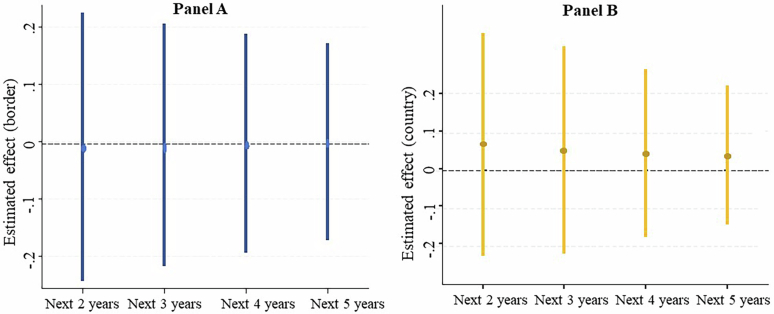
Placebo tests with future policy effects (difference in discontinuities and difference in differences). Panel **a** indicates the placebo tests of the effects of future policies (next 2 years to next 5 years) on cropland condition measurements. The policy variable is a normal (non-weighted) policy dummy taking the value of one if the country has more policies and zero otherwise. To determine the policy leads, we take forward the values of policy variables as an opposite way to taking lags. Then, the policy effects are estimated using a difference in discontinuity design for the border regions. Each estimates show insignificant effect implying that the parallel trend assumption hold. Panel **b**: This panel indicates similar policy measures like in panel **b**, but the estimates are from difference in differences. In both placebo tests, we obtain insignificant effects, confirming that our estimates are unlikely to be biased and that parallel trend assumptions hold. For panel **a**, the sample size is about 83 million pixel-year observations, whereas for the panel **b** the sample is country average values (N = 3,040). Error bars represent 95% confidence intervals. See Supplementary Fig. 1 for further information. Source data

Source Data Extended Data Fig. 4 Source data for Extended Data Fig. 4.

**Extended Data Fig. 5:**
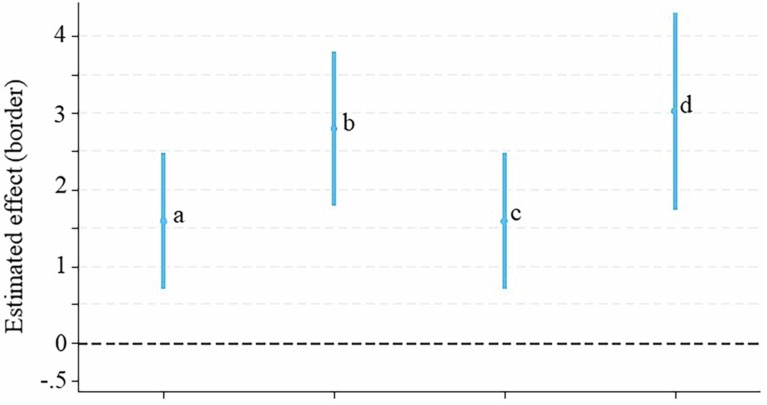
Placebo tests with border discontinuity (border difference in discontinuities). The figure shows a robustness check for border endogeneity. This is done by limiting our difference-in-discontinuity analysis to only those international borders with no natural productivity discontinuities. This finding confirms that our finding is robust to the potential spurious endogeneity from the border location. Sample size corresponds to ~83 million grid-cell–year observations. Error bars represent 95% confidence intervals. Source data

Source Data Extended Data Fig. 5 Source data for Extended Data Fig. 5.

**Extended Data Fig. 6:**
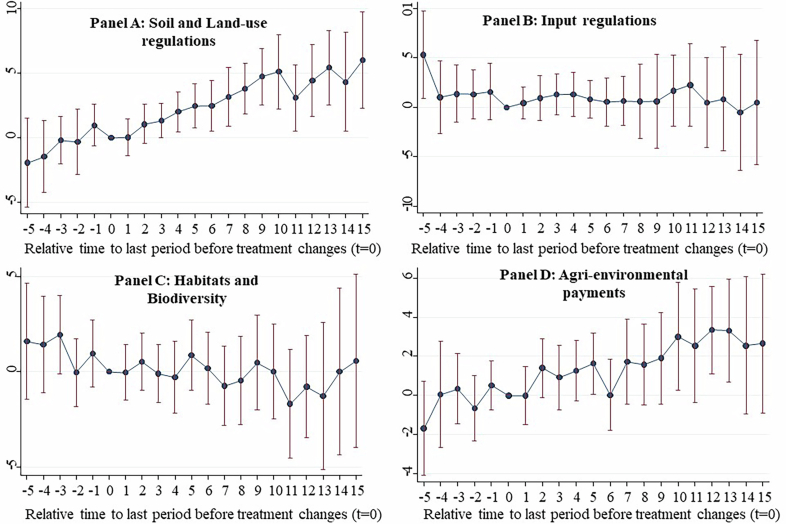
Dynamic event-study estimates of policy heterogeneity across policy types. This figure corresponds to the dynamic effect of policy heterogeneity presented in Fig. 5, but now as an event study with a clear pre-trend. As shown in panels, the event study is for the four different policy types: (**a**) soil and land use regulations, (**b**) input regulations, (**c**) habitats & biodiversity regulations, and (**d**) agri-environmental payments. The estimates are obtained using the staggered event study estimator and clearly show the pre-trends of policy effect, which implies that parallel trends hold. Each types of policy are directly counted to capture variation in policy intensity across policy types. Sample size (N = 3,040) corresponds to country–year observations (biological replicates), with grid-cell measurements averaged within country-years (technical replicates). Error bars represent 95% confidence intervals. Source data

Source Data Extended Data Fig. 6 Source data for Extended Data Fig. 6.

## Data Availability

Source data are provided with this paper. All other data are available via Zenodo at 10.5281/zenodo.19480959 (ref. ^74^) and from the corresponding author upon reasonable request^53,56,57,75–77^.

## Code availability

All code and programs are available via Zenodo at 10.5281/zenodo.19480959 (ref. ^74^) and from the corresponding author upon reasonable request.
