# Supplementary material for: Agri-environmental policies have reduced cropland degradation globally
Source: Nat Food. 2026 May 18;7(6):527–38. doi: 10.1038/s43016-026-01359-4 (PMC13290496; doi:10.1038/s43016-026-01359-4)
Supplement: Supplementary file 1 — Supplementary Figs. 1–9, Tables 1–5 and Notes, including legends for all figures and tables. [file 43016_2026_1359_MOESM1_ESM.pdf]

nature food

Supplementary information https://doi.org/10.1038/s43016-026-01359-4

Agri-environmental policies have reduced cropland degradation globally

In the format provided by the authors and unedited

Supplementary Information

Supplementary Figure 1: Public policies and relevant country contextual indicators

Supplementary Figure 2: Illustrative examples of discontinuities in cropland condition

Supplementary Figure 3: Robustness analysis without management practices

Supplementary Figure 4: Trends in cropland condition and total factor productivity

Supplementary Figure 5: Trends in cropland condition and crop yield

Supplementary Figure 6: Treatment status of countries by time periods and regions

Supplementary Figure 7: Robustness with controls for crop types

Supplementary Figure 8: Threshold-based dynamic estimates of policy effects

Supplementary Figure 9: Lag effects of policies

Supplementary Table 1: Dynamic estimates of policy effects

Supplementary Table 2: Examples of public agri-environmental policies

Supplementary Table 3: Descriptions of all variables at the grid-cell and country level

Supplementary Table 4: Summary statistics of all variables, at grid and country level

Supplementary Table 5: Border discontinuities time-varying covariate balance test

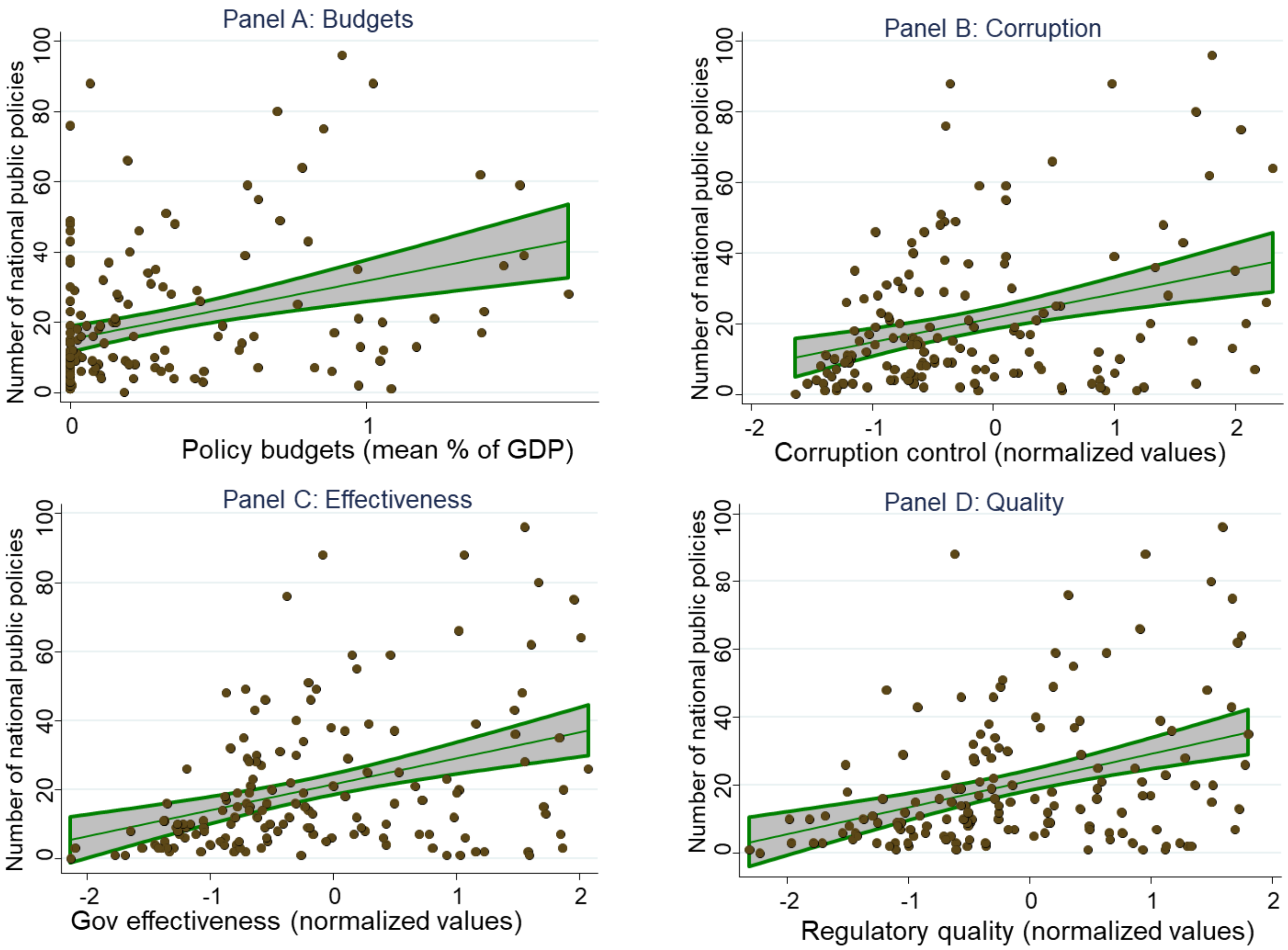

Supplementary Figure 1. Public policies and relevant country contextual indicators. These figures illustrate the relationship between the total number of public policies implemented by countries and other relevant policy indicators. Panel A shows the relationship between public policy budgets (government expenditure on environment protection) and the number of public policies, implying that countries with more policies tend to allocate larger budgets to environmental policy design and implementation. Panel B shows that countries with a higher number of public policies are also more likely to have corruption control measures. Similarly, Panels C and D show that countries with a greater number of policies generally exhibit higher government effectiveness and regulatory quality, which are essential for effective policy design and implementation. The dark dots represent countries.

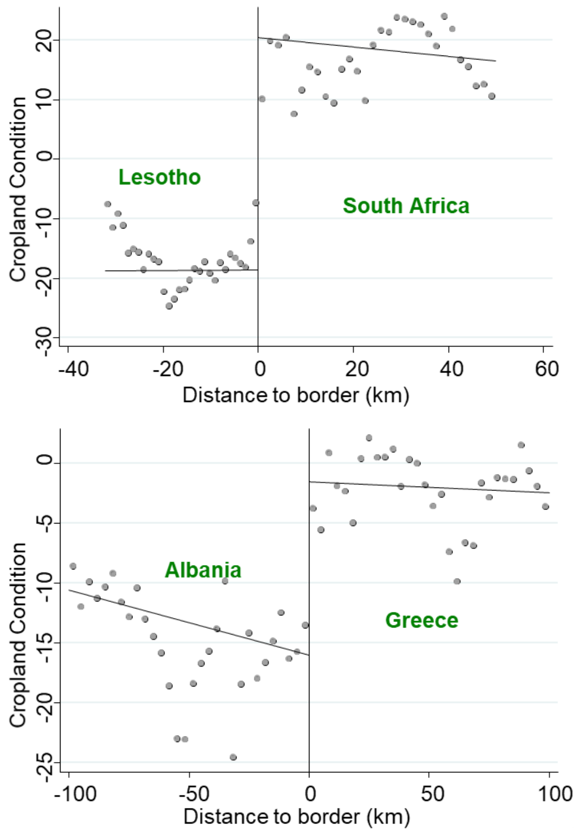

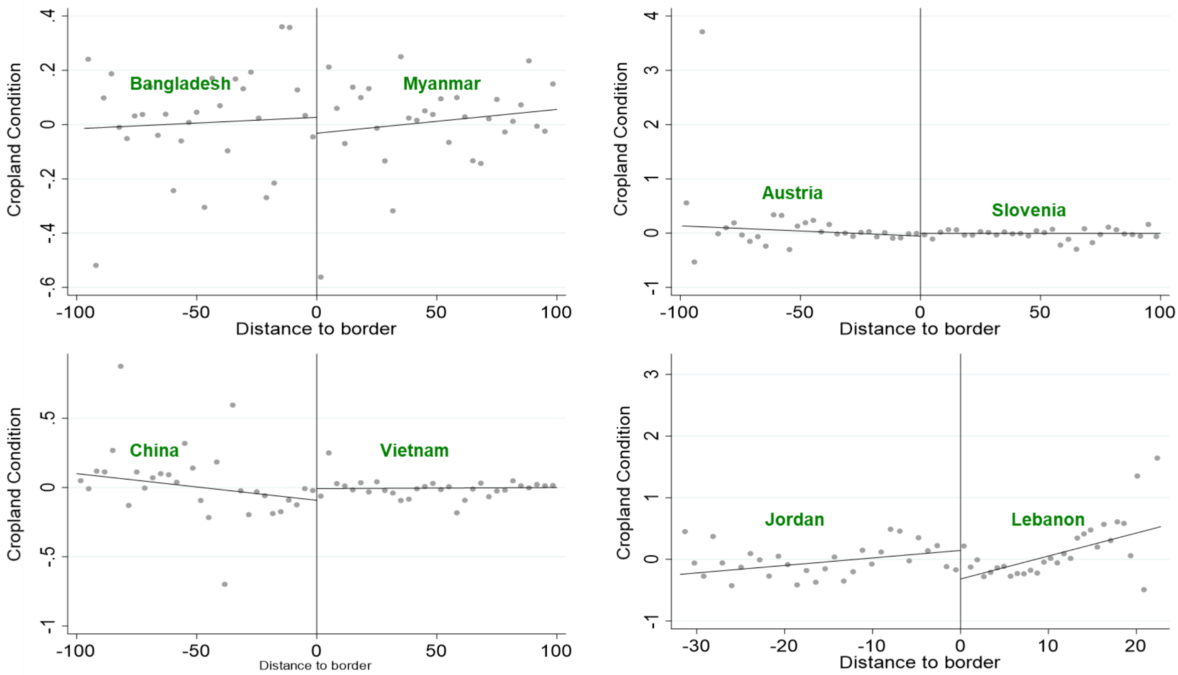

Supplementary Figure 2. Illustrative examples of discontinuities in cropland condition. The figure shows examples of border discontinuities in cropland condition between pairs of country borders: Lesotho-South Africa, Cambodia-Vietnam, Albania-Greece, Iran-Azerbaijan, Bangladesh-Myanmar, Austria-Slovenia, China-Vietnam, and Jordan-Lebanon. It indicates the spatial distribution of measured cropland condition within 100 km of each border. The distance to the border represents the normalized distance of the crop pixel in kilometers running from the border points in both sides. The negative distance on the left represents countries with fewer public policies

(Lesotho, Cambodia, Albania, Iran, Bangladesh, Austria, China, and Jordan), while the positive distance on the right represents countries with more policies (South Africa, Vietnam, Greece, Azerbaijan, Myanmmar, Slovenia, Vietnam and Lebanon). The black dots are local average values of cropland measure, while the lines are fitted spatial trends. The dotted vertical line is the average pair-county border. Sample size is drawn from ~83 million grid-cell–year observations from border regions.

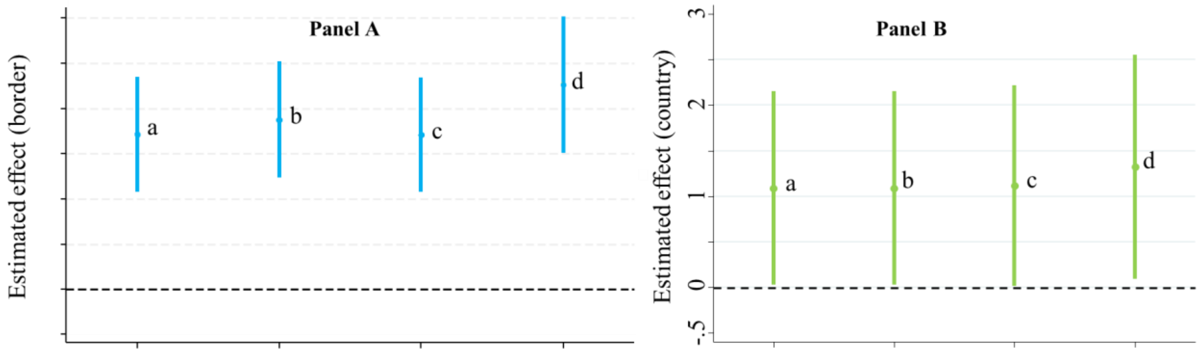

Supplementary Figure 3. Robustness analysis without management practices (difference in discontinuities and difference in differences). The figure corresponds to the main result of Figure 2, but without controlling for management practices (irrigation, fertilizer, pesticide and tillage) in equation 1. Like figure 2, lowercase letters (a-i) show different policy measures: a) policy without weight; b) government effectiveness weighted; c) environmental expenditure weighted policy; and; i) sum of all weighted policy variables. For panel A, sample size corresponds to ~83 million grid-cell–year observations, and for panel B, it is about 3,040 country-year observations. Error bars represent 95% confidence intervals.

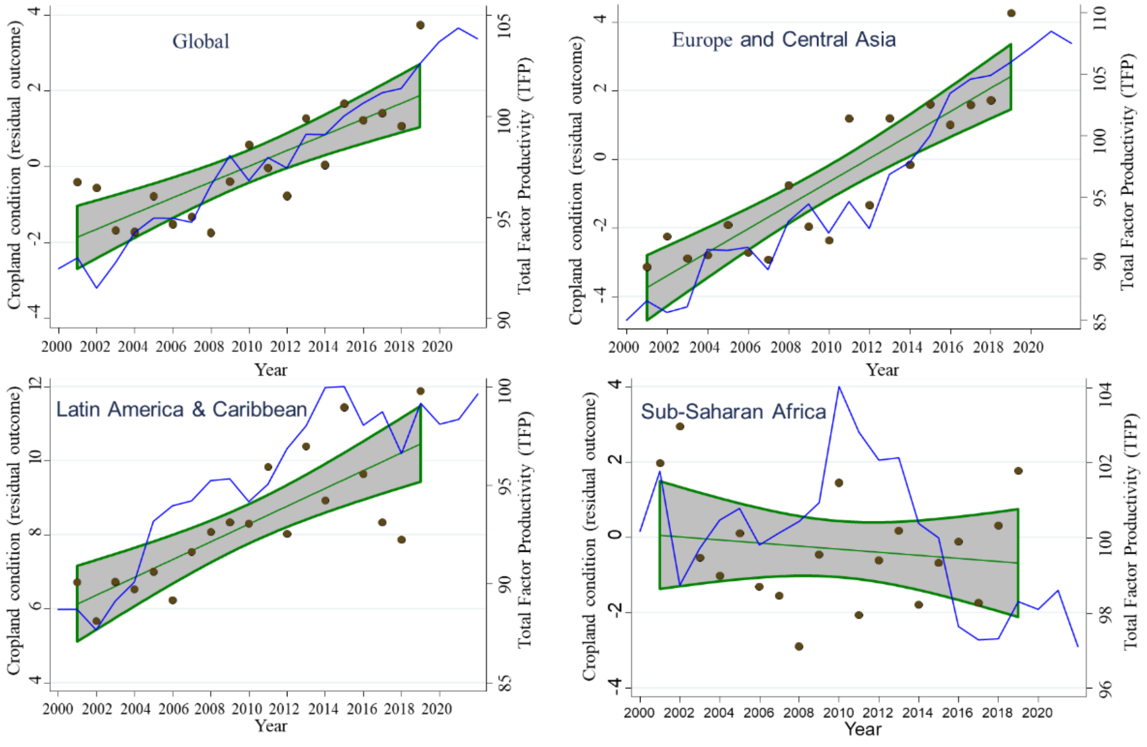

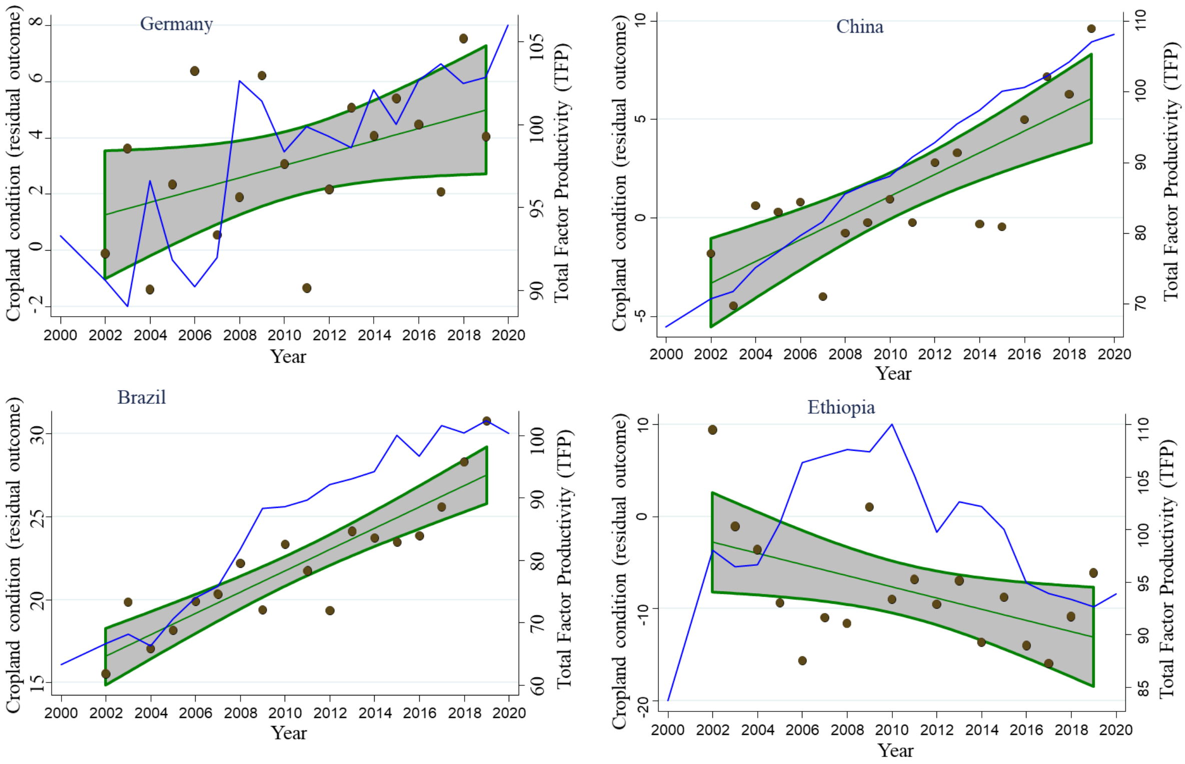

Supplementary Figure 4. Trends in cropland condition and total factor productivity (TFP). The figure presents trends in cropland condition and total factor productivity (TFP) over time for the global level, selected regions (Europe and Central Asia, Latin America and the Caribbean, and Sub-Saharan Africa), and four countries (Germany, China, Brazil and Ethiopia). The x-axis represents time (years), while the y-axis shows standardized values of cropland condition (brown dots) and TFP (blue line). Cropland condition is plotted with a fitted linear trend (green line) and 95% confidence interval (shaded area). At global level and across most regions, cropland condition and TFP exhibit a clear upward trajectory, indicating that improvements in land quality are associated with gains in total factor productivity. However, in Sub-Saharan Africa, cropland condition shows a modest overall decline, while TFP exhibits a spike in earlier years followed by a decline, highlighting a partial divergence in trends. At the country level, Germany, China, and Brazil show consistent upward trends in both measures, whereas Ethiopia displays a downward trend in cropland condition and a volatile TFP pattern with a declining trajectory in recent years.

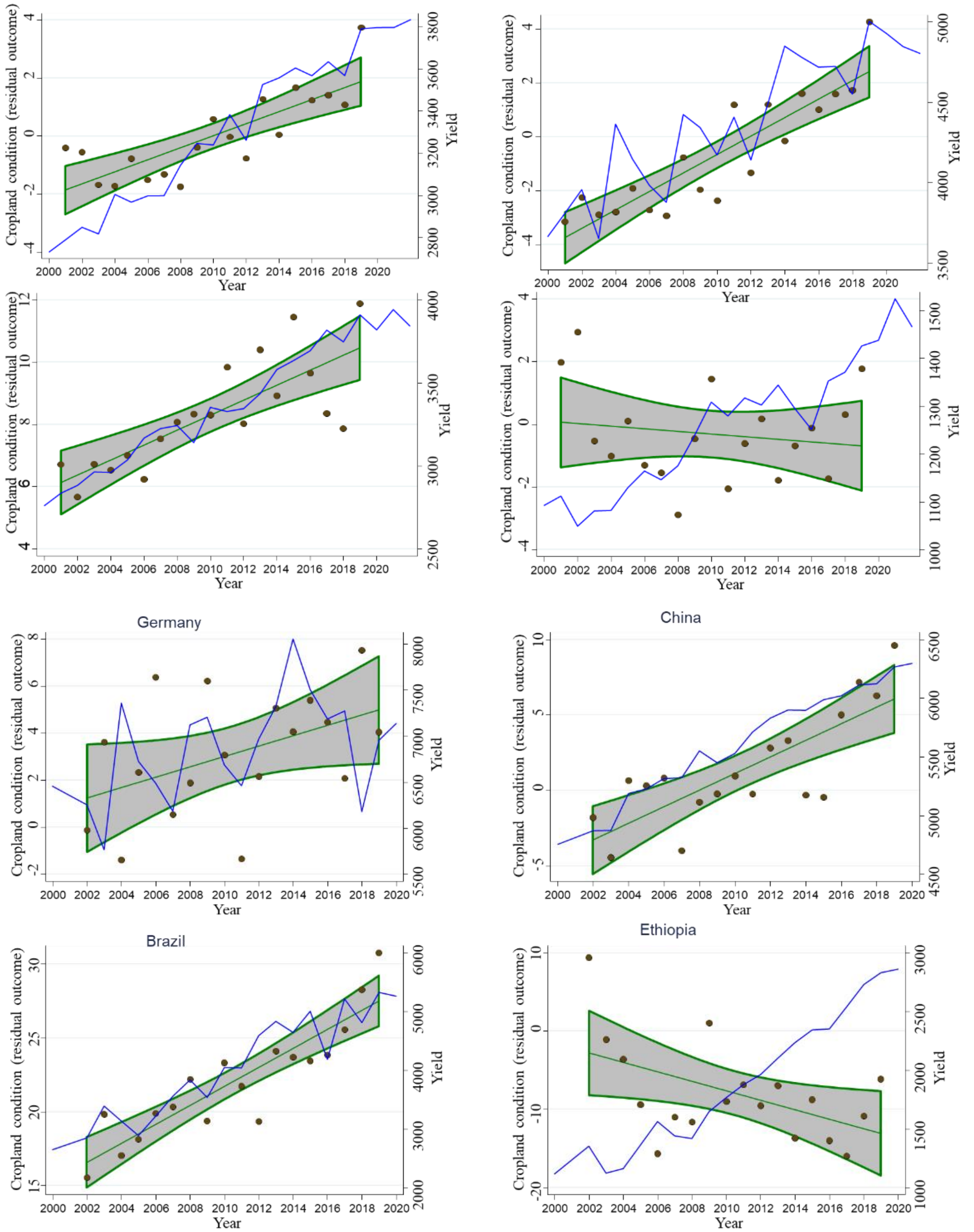

Supplementary Figure 5. Trends in cropland condition and crop yield. The figure presents trends in cropland condition and yield over time for the global level, selected regions (Europe and Central Asia, Latin America and the Caribbean, and Sub-Saharan Africa), and four countries (Germany, China, Brazil and Ethiopia). The x-axis represents time (years), while the y-axis shows standardized values of cropland condition (brown dots) and Yield (blue line). Cropland condition is plotted with a fitted linear trend (green line) and 95% confidence interval (shaded area). At global level and across most regions, cropland condition and yield exhibit a clear upward trajectory, indicating that improvements in land quality are associated with increased yield. However, in Sub-

Saharan Africa, cropland condition shows a modest overall decline, while yield exhibits overall increases, highlighting a divergence in trends. At the country level, Germany, China, and Brazil show consistent upward trends in both measures, whereas Ethiopia displays a downward trend in cropland condition but steadily increasing yield pattern with a sharp trajectory in later post 2010 years.

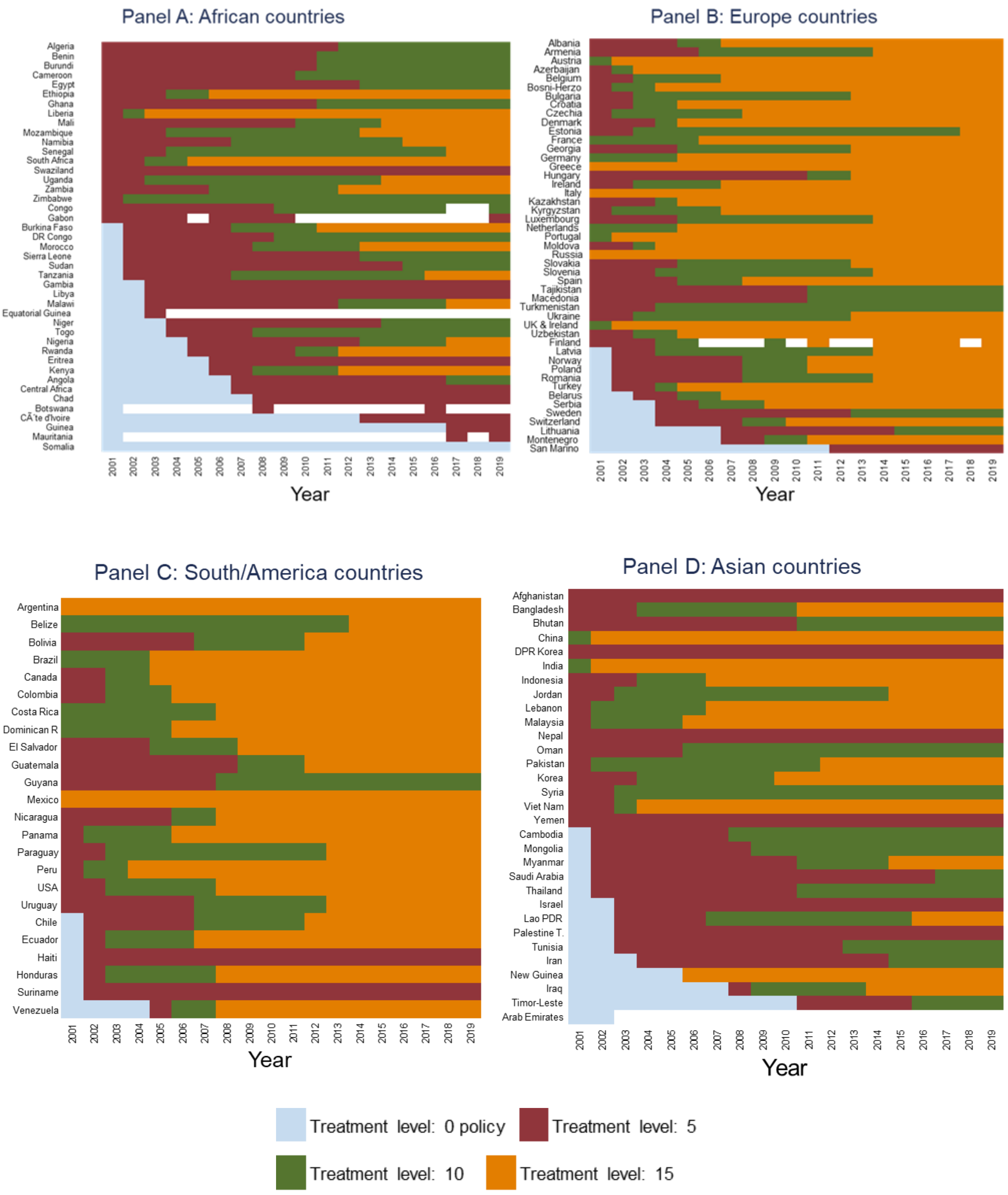

Supplementary Figure 6. Treatment status of countries by time periods and regions. The figure presents different thresholds of public policies for different regions over the years. Panel A is for African countries. Panel B is for European countries. Panel C is for the South and North American countries. Panel D is for Asian countries.

As shown in the legend, the first light blue threshold shows zero number of policies. This is followed by a number of policies greater than zero, but less than 5, shown by red. Then, the green box refers to the number of policies between 5 and 10. Finally, dark orange indicates the number of policies greater than 10.

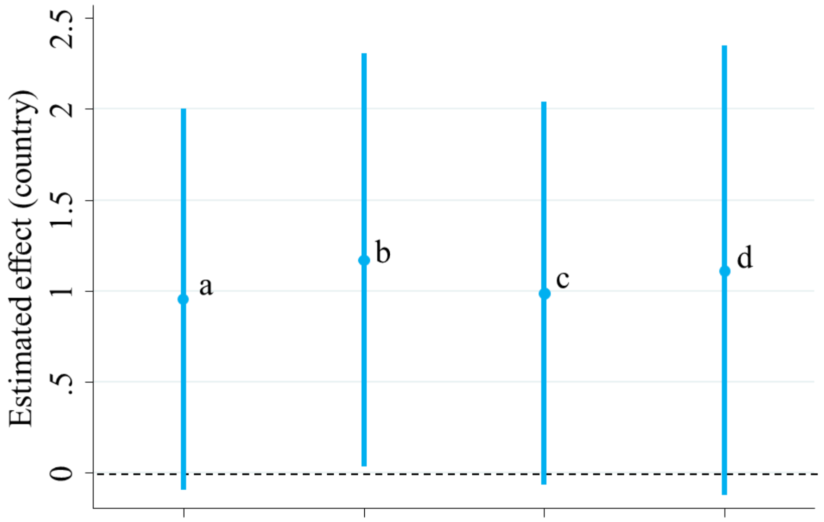

Supplementary Figure 7. Robustness checks with controls for crop types (difference in differences). This figure corresponds to the main result of Figure 2, but with additionally controlling dominant crop types, using aggregated country-level crop distribution data (for top 10 crops)[68]. Our results remain robust to this adjustment. Sample size corresponds to 3,040 country-year observations. Error bars represent 95% confidence intervals.

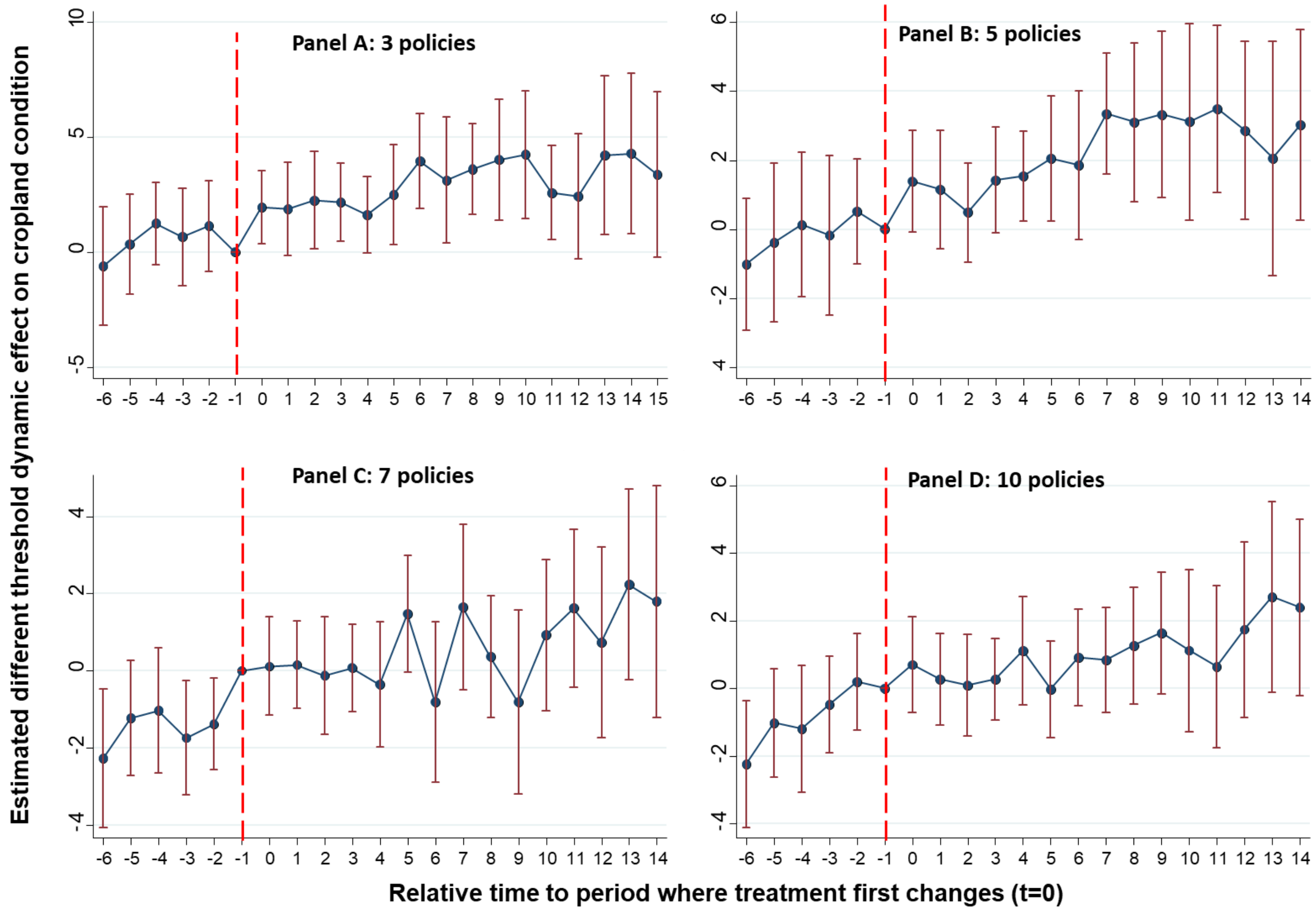

Supplementary Figure 8. Threshold-based dynamic estimates of the effects of policies on cropland condition. This figure corresponds to the dynamic effect figure 3, but with different policy thresholds. In panels A, B, C and D, we choose that country with at least 3, 5, 7 and 10 public policies are sorted into the treatment group, whereas others are sorted into the control group. Our result shows similar patterns to figure 3, but the overall effect is in decreasing order as we increase the thresholds. The average effect with at least 3 policies is 2.9%, with at least 5 policies is 2%, with at least 7 policies is 0.43% and with at least 10% is about 0.80%. The overall effect is only statistically significant at the threshold of 3 and 5 policy thresholds. This robustness highlights that by choosing different policy thresholds, one can see that the obtained effect is not just driven by the specific threshold chosen for analysis. Moreover, we observe a larger policy effect when comparing countries with relatively fewer policies with relatively more policies when the threshold is low. Sample size (N = 3,040) corresponds to country–year observations (biological replicates), with grid-cell measurements averaged within country-years (technical replicates). Error bars represent 95% confidence intervals.

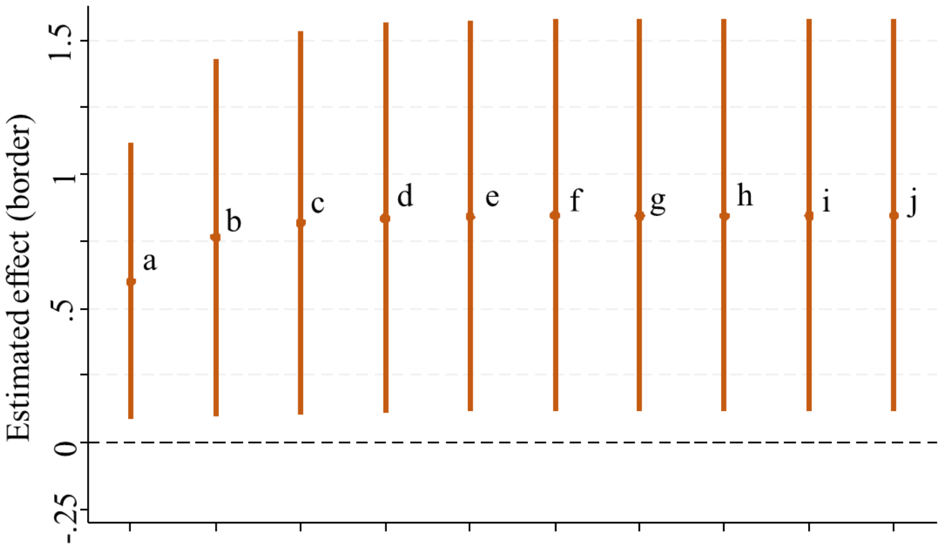

Supplementary Figure 9. Lag effects of policies (difference in discontinuities). This figure indicates the estimated effect of lagged policies on cropland condition using difference in discontinuities. The lags of policy variable are determined by taking lag of policy variable. Y-axis are coefficients of lagged policy variables. The corresponding small letters (a-i) in X-axis show different policy measures different policy lags, (a) 1 year, (b) two years and up to (j) 10 years lag. Our estimates show that the estimated policy effect steadily increases over time for the initial 6 years, after which it plateaus, with no further increase in effect size observed. This gives us an implication that the estimated policy effect is cumulative. Sample size corresponds to ~83 million grid-cell–year observations. Error bars represent 95% confidence intervals.

Supplementary Table 1. Dynamic estimates of the effects of policies on cropland

Panel A: Causal estimates

Post period	Estimate	SE	LB CI	UB CI
0	-1.851257	1.506911	-4.804802	1.102289
1	1.980216	1.564527	-1.086258	5.04669
2	4.018462	1.844488	0.4032654	7.633659
3	4.855729	2.217295	0.5098318	9.201626
4	4.18011	2.083287	0.0968678	8.263353
5	2.013645	2.215336	-2.328415	6.355704
6	2.770333	2.687782	-2.49772	8.038385
7	-1.129163	2.01361	-5.075838	2.817513
8	4.481501	2.406026	-0.2343107	9.197312
9	5.526146	1.978623	1.648045	9.404248
10	2.910151	1.56701	-0.1611884	5.98149

11	6.901831	2.568439	1.867692	11.93597
12	8.017584	3.132997	1.876909	14.15826
13	9.720241	4.099304	1.685606	17.75488
14	9.412091	4.489928	0.6118313	18.21235
15	10.42611	4.112166	2.366268	18.48596
Average	4.420854	1.560139	1.36298	7.478727

Panel B: Placebo estimates

Pre-period	Estimate	SE	LB CI	UB CI
1	0.3628741	2.35769	-4.258199	4.983947
2	-1.50974	2.788123	-6.974461	3.95498
3	-0.7818565	4.30916	-9.22781	7.664097
4	3.724935	4.635631	-5.360901	12.81077
5	3.581922	5.327098	-6.859191	14.02304

Supplementary Table 2. Examples of public agri-environmental policies

Category	Area	Country Example	Short description of policies
Legislation	Agriculture	Kenya (2012) National legislative Act	It lays down rules on good agricultural practice and, in particular, on the development and conservation of agricultural land. It also provides administrative instruments for the sound development of agriculture and the marketing of agricultural products.
Legislation	Agriculture	South Korea (2015) National legislation	It regulates planning, protection, control, care and proper use of agricultural land and soil with supervision for correct application.
Legislation	Agriculture	Kyrgystan (2012) National legislation	It regulates relations in the sphere of protection of soil, fertility, conservation of quality and protection against degradation and other negative impact on agricultural land.
Payment Scheme	Agriculture	Turkey (2006) Environmental Based Agricultural Protection Program	It protects water and soil quality, native vegetation, and prevents soil and wind erosion, reduces crop surpluses, applies alternative crop production models, and promotes farming practices that preserve and protect the environment.
Payment Scheme	Agriculture	Taiwan (2018) Green Environmental Payment Program	It encourages eco-friendly farming practices that improve the quality of crops, and ensure farmland devoted to agricultural use. It also aims to promote sustainable development agricultural industry while benefiting the farmers.

Payment Scheme	Forest/ Agriculture	China (2018) Grain for Green Program	It pays farmers to plant trees on their land and provides degraded land to rural families to restore to reduce soil erosion and flooding while alleviating rural poverty.
Monitoring/ framework	Biodiversity	Barbados (2002) National Biodiversity Strategy and Action Plan	It ensures the conservation and sustainable use of biological diversity while addressing intensive agriculture that contributed to erosion of topsoil, decrease in soil fertility, and large inputs of pesticides and chemical fertilizers.
Monitoring/ framework	Agriculture	Brazil (2009) Legislation	It protects soil quality and manages contaminated areas to guarantee the sustainable use of the soil, avoiding its contamination and preventing ecosystem changes in its characteristics and functions, including protecting the quality of soil and groundwater.
Monitoring/ framework	Fertilizer	China (2010) Strategy Act	It aims to prevent and remediate soil and groundwater pollution, ensure the sustainable use of soil and groundwater, and enhance the living environment. It also applies regular monitoring of the quality of the soil and groundwater, and verification of sites suspected of having soil and groundwater pollution, and control of pollution sources.

Supplementary Table 3. Descriptions of all variables at the grid-cell and country level

Variable	Descriptions	Data source
Enhanced Vegetation index (EVI)	Quantifies year-to-year changes in annual maximum vegetation greenness at the grid cell level during the period of 2001-2019 (for 83 million pixel-year observations from all around the world).	Didan & Munoz [28]
Cropland condition (border regions)	Soil condition measurements estimated based on the reading of annual maximum EVI at grid cell level by accounting for climatic and human management factors.	
Average cropland condition (entire country)	Averaged cropland soil condition measurements from the reading of annual maximum EVI at country level	
Total precipitation (mm)	Annual average rainfall data at the grid-cell level during the years 2001-2019.	Schneider et al. [52]
Land surface temperature (Celsius)	Annual average land surface temperature at the grid-cell level during the years 2001-2019.	Wan [53]

Elevations (meters)	Elevation at the grid-cell level (at 90 meters)	Robinson et al. [55]
Solar radiation	Annual average solar radiation at the grid-cell level during the years 2001-2019.	Tang et al. [54]
Irrigation	Average irrigation at the grid-cell level for year 2005	Siebert et al. [57]
Synthetic nitrogen fertilizer	Synthetic nitrogen fertilizer application rates on cropland at the grid-cell level (5 arcmin resolution) yearly from 2001-2019	Tian et al. 2022. [56]
Share of pixel cultivated	Share of cultivated cropland at the grid-cell level during year 2000	Didan & Munoz [28]
Agri-environmental policies	The cumulative sum of all countries soil related public policies since year 2000 at the country level, including legislative changes, new regulations, payments for ecosystem services, policy reforms, monitoring	Wuepper et al. [10]
Property rights index (0-100)	Measure of economic institutions at the country level, reflecting the degree to which a country's laws protect private property rights and the degree to which those laws are enforced	Heritage Foundation [76]
Human development index (HDI)	The summary measure of average achievement in 3 key dimensions of human development at the country level: a long and healthy life, being knowledgeable and having a decent standard of living	
GDP per capita	Annual gross domestic product per capita (current and constant in 2010 USD) at the country level.	World bank [77]
GDP share agriculture	The contribution of the agricultural sector to GDP at the country level	World bank [77]
Environmental expenditure	Each government yearly expenditure (during 2001-2020) at the country level for protection of environment, including pollution abatement, waste management, research and development	IMF [35]
Control of corruption	Captures the perceptions of the extent to which public power is exercised for private gain including diversion of funds, irregular payments, officials/local administrative corruptions at the country level	World bank [34]
Government effectiveness	Measures the perceptions of the quality of public services and the degree of its independence from the political pressures, including the quality	World Bank [34]

	of policy and creditability of the government's commitment to their policies at the country level	
Regulatory quality	Captures the perceptions of the ability of the government to formulate and implement sound policies and regulations that permit and promote private sector development at the country level	World Bank [34]
Political stability and absence of terrorism/violence	Measures perceptions of the likelihood of political instability and/or politically motivated violence including terrorism at the country level	World Bank [34]
Rule of Law	Captures perceptions of the extent to which agents have confidence in and abide by the rules of the society, including the enforcement, property rights, the police and courts at the country level	World Bank [34]
Voice and Accountability	Measures perceptions of the extent to which a country's citizens are able to participate in selecting their government, as well as freedom of expression, freedom of association, and a free media at the country level	World Bank [34]

Supplementary Table 4. Summary statistics of all variables, both at grid and country levels

Variable	(1) observation	(2) Fewer policies	(3) More policies
**Grid cell level**			
Log cropland condition	79,003,508	-0.006	0.005
		(0.197)	(0.193)
Cropland condition	79,003,517	-24.992	20.569
		(940.931)	(944.706)
Log of maximum EVI	81,258,755	8.527	8.546
		(0.245)	(0.222)
Maximum EVI	81,258,792	5,193.025	5,267.434
		(1,150.982)	(1,080.573)
Total precipitation (mm)	81,258,881	860.729	850.136
		(591.494)	(566.554)
Land surface temperature (Celsius)	80,926,904	17.307	16.172
		(8.413)	(8.724)
Elevations (meters)	81,258,881	338.250	357.044
		(373.939)	(443.991)

Solar radiation	80,698,986	127.125	126.544
		(17.368)	(17.379)
Irrigation	81,258,881	855.145	926.230
		(1,649.857)	(1,735.767)
Soil nitrogen content	79,870,637	403.665	411.948
		(292.601)	(284.557)
Soil organic carbon	79,860,070	383.072	409.234
		(257.553)	(260.619)
share of pixel cultivated (percent) in 2000	81,258,881	80.449	80.563
		(22.153)	(22.520)
**Country level**			
all public agri-environmental policies	3,040	10.301	28.779
		(10.710)	(31.975)
Bayesian corruption	3,040	52.971	47.999
		(12.495)	(12.939)
Environmental expenditure	3,040	0.964	1.253
		(0.738)	(0.778)
Property rights protection	3,040	50.854	51.767
		(3.088)	(2.973)
Control of corruption	3,040	-0.474	-0.231
		(0.876)	(0.890)
Government effectiveness	3,040	-0.287	-0.015
		(0.892)	(0.828)
Political stability and terrorism	3,040	-0.505	-0.423
		(0.997)	(0.920)
Regulatory quality	3,040	-0.212	-0.025
		(0.921)	(0.867)
Rule of Law	3,040	-0.369	-0.124
		(0.918)	(0.879)
Voice and accountability	3,040	-0.294	-0.134
		(0.978)	(0.987)

Supplementary Table 5. RDD time-varying covariate balance test

	**Treatment**			**Control**			**Balance**	
	N	Mean	SD	N	Mean	SD	SMD	VR
Solar Radiation	20,009,810	126.80	17.26	22,028,188	126.74	16.95	0.00	1.04
Precipitation	20,009,810	866.07	605.92	22,028,188	853.95	604.29	0.02	1.01
Temperature	20,009,810	16.68	8.66	22,028,188	16.68	8.56	0.00	1.02
Altitude	20,009,810	334.95	405.57	22,028,188	328.25	386.46	0.02	1.10

Irrigation	20,009,810	906.34	1697.53	22,028,188	894.93	1658.41	0.01	1.05
Soil Nitrogen	20,009,810	407.86	285.74	22,028,188	407.00	286.36	0.00	1.00
Organic Carbon	20,009,810	397.99	261.31	22,028,188	393.48	259.38	0.02	1.01

Notes: The table shows the standard covariate balance test across the international border. The test results indicate that there are no significant differences between the treated (more policy) and control (fewer policy) groups in terms of the covariates measured (solar radiation, precipitation, temperature, altitude, irrigation, soil nitrogen, and soil organic carbon). The standardized mean difference (SMD) values are all very close to 0, and the variance variation (VR) values are close to 1, indicating balanced covariates across the treatment threshold.
