# Supplementary material for: Agri-environmental policies have reduced cropland degradation globally
Source: Nat Food. 2026 May 18;7(6):527–38. doi: 10.1038/s43016-026-01359-4 (PMC13290496; doi:10.1038/s43016-026-01359-4)
Supplement: Supplementary file 2 — Reporting Summary [file 43016_2026_1359_MOESM2_ESM.pdf]

nature portfolio

Corresponding author(s): DBRPR -NATFOOD-24091824B

Last updated by author(s): Apr 10, 2026

Reporting Summary

Nature Portfolio wishes to improve the reproducibility of the work that we publish. This form provides structure for consistency and transparency in reporting. For further information on Nature Portfolio policies, see our Editorial Policies and the Editorial Policy Checklist.

Statistics

For all statistical analyses, confirm that the following items are present in the figure legend, table legend, main text, or Methods section.

n/a	Confirmed	
☐	☒	The exact sample size (*n*) for each experimental group/condition, given as a discrete number and unit of measurement
☐	☒	A statement on whether measurements were taken from distinct samples or whether the same sample was measured repeatedly
☐	☒	The statistical test(s) used AND whether they are one- or two-sided<br>*Only common tests should be described solely by name; describe more complex techniques in the Methods section.*
☐	☒	A description of all covariates tested
☐	☒	A description of any assumptions or corrections, such as tests of normality and adjustment for multiple comparisons
☐	☒	A full description of the statistical parameters including central tendency (e.g. means) or other basic estimates (e.g. regression coefficient) AND variation (e.g. standard deviation) or associated estimates of uncertainty (e.g. confidence intervals)
☐	☒	For null hypothesis testing, the test statistic (e.g. F, t, r) with confidence intervals, effect sizes, degrees of freedom and P value noted<br>*Give P values as exact values whenever suitable.*
☒	☐	For Bayesian analysis, information on the choice of priors and Markov chain Monte Carlo settings
☐	☒	For hierarchical and complex designs, identification of the appropriate level for tests and full reporting of outcomes
☒	☐	Estimates of effect sizes (e.g. Cohen's *d*, Pearson's *r*), indicating how they were calculated

Our web collection on statistics for biologists contains articles on many of the points above.

Software and code

Policy information about availability of computer code

Data collection	Our data collection process involves leveraging the recently published public policy database by Wuepper et al. (2024, available at (at https://www.nature.com/articles/s43016-024-00945-8) with satellite data for measuring cropland condition. We identify global cropland areas using the data provided by Sulla-Menashe et al. (2019). All variables including climate and country characteristics variables are explained in the manuscript, including their sources. Overall, our analysis incorporates approximately 2,700 public agri-environmental policies and about 83 million cropland pixels spanning the years from 2000 to 2019. For satellite data preparation, we utilized Google Earth Engine and R software.
Data analysis	Our empirical analysis employs two complementary econometric techniques: difference-in-discontinuities and difference-in-differences. We used stata 18 Software for the analysis.

For manuscripts utilizing custom algorithms or software that are central to the research but not yet described in published literature, software must be made available to editors and reviewers. We strongly encourage code deposition in a community repository (e.g. GitHub). See the Nature Portfolio guidelines for submitting code & software for further information.

Data

Policy information about availability of data

All manuscripts must include a data availability statement. This statement should provide the following information, where applicable:

- Accession codes, unique identifiers, or web links for publicly available datasets
- A description of any restrictions on data availability
- For clinical datasets or third party data, please ensure that the statement adheres to our policy

The public policy database is accessible (at https://www.nature.com/articles/s43016-024-00945-8). All covariates used in the analysis, including climate and country-level characteristic variables, are thoroughly documented in the manuscript. The code used for econometric analysis is under preparation and soon published online. We are committed to depositing data and code in open community repository, prior to publication to enhance the transparency and reproducibility of our findings.

Research involving human participants, their data, or biological material

Policy information about studies with human participants or human data. See also policy information about sex, gender (identity/presentation), and sexual orientation and race, ethnicity and racism.

Reporting on sex and gender	No human research participants involved in this study
Reporting on race, ethnicity, or other socially relevant groupings	N/A
Population characteristics	N/A
Recruitment	N/A
Ethics oversight	N/A

Note that full information on the approval of the study protocol must also be provided in the manuscript.

Field-specific reporting

Please select the one below that is the best fit for your research. If you are not sure, read the appropriate sections before making your selection.

☐ Life sciences ☐ Behavioural & social sciences ☒ Ecological, evolutionary & environmental sciences

For a reference copy of the document with all sections, see nature.com/documents/nr-reporting-summary-flat.pdf

Ecological, evolutionary & environmental sciences study design

All studies must disclose on these points even when the disclosure is negative.

Study description	The study globally analyzes the impact of public agri-environmental policies on the state of cropland. We employ two complementary quasi-experimental econometric techniques: differences in discontinuities and differences in differences.
Research sample	We analyze approximately 83 million measurements of cropland pixels and around 2,700 public agri-environmental policies.
Sampling strategy	N/A
Data collection	The public policy database is accessed from publicly available data (at https://www.nature.com/articles/s43016-024-00945-8). Satellite data are sourced from open-access platforms, with detailed information documented in the manuscript
Timing and spatial scale	The pixel-level data is gap-free at a fine resolution of 1x1 km, covering the period from 2001 to 2019. The public policy database also spans the same timeframe.
Data exclusions	N/A
Reproducibility	All data analyses are reproducible, and all codes and data are available in the manuscript and stored in public repository.
Randomization	N/A
Blinding	N/A

Did the study involve field work? ☐ Yes ☒ No

Reporting for specific materials, systems and methods

We require information from authors about some types of materials, experimental systems and methods used in many studies. Here, indicate whether each material, system or method listed is relevant to your study. If you are not sure if a list item applies to your research, read the appropriate section before selecting a response.

Materials & experimental systems

n/a	Involved in the study
☒	☐ Antibodies
☒	☐ Eukaryotic cell lines
☒	☐ Palaeontology and archaeology
☒	☐ Animals and other organisms
☒	☐ Clinical data
☒	☐ Dual use research of concern
☒	☐ Plants

Methods

n/a	Involved in the study
☒	☐ ChIP-seq
☒	☐ Flow cytometry
☒	☐ MRI-based neuroimaging

Plants

Seed stocks	n/a
Novel plant genotypes	n/a
Authentication	n/a
